# A Barcode Screen for Epigenetic Regulators Reveals a Role for the NuB4/HAT-B Histone Acetyltransferase Complex in Histone Turnover

**DOI:** 10.1371/journal.pgen.1002284

**Published:** 2011-10-06

**Authors:** Kitty F. Verzijlbergen, Tibor van Welsem, Daoud Sie, Tineke L. Lenstra, Daniel J. Turner, Frank C. P. Holstege, Ron M. Kerkhoven, Fred van Leeuwen

**Affiliations:** 1 Department of Gene Regulation, Netherlands Cancer Institute, Amsterdam, The Netherlands; 2 Genome Center, Netherlands Cancer Institute, Amsterdam, The Netherlands; 3 Netherlands Proteomics Center, Amsterdam, The Netherlands; 4 Department of Molecular Cancer Research, University Medical Center Utrecht, Utrecht, The Netherlands; 5 Wellcome Trust Sanger Institute, Wellcome Trust Genome Campus, Hinxton, United Kingdom; Friedrich Miescher Institute for Biomedical Research, Switzerland

## Abstract

Dynamic modification of histone proteins plays a key role in regulating gene expression. However, histones themselves can also be dynamic, which potentially affects the stability of histone modifications. To determine the molecular mechanisms of histone turnover, we developed a parallel screening method for epigenetic regulators by analyzing chromatin states on DNA barcodes. Histone turnover was quantified by employing a genetic pulse-chase technique called RITE, which was combined with chromatin immunoprecipitation and high-throughput sequencing. In this screen, the NuB4/HAT-B complex, containing the conserved type B histone acetyltransferase Hat1, was found to promote histone turnover. Unexpectedly, the three members of this complex could be functionally separated from each other as well as from the known interacting factor and histone chaperone Asf1. Thus, systematic and direct interrogation of chromatin structure on DNA barcodes can lead to the discovery of genes and pathways involved in chromatin modification and dynamics.

## Author Summary

Packaging of eukaryotic genomes by the histone proteins influences many processes that use the DNA, such as transcription, repair, and replication. One well-known mechanism of regulation of histone function is the covalent modification of histone proteins. Replacement of modified histones by new histones has recently emerged as an additional layer of regulation (hereafter referred to as histone turnover). Although histone replacement can affect substantial parts of eukaryotic genomes, the mechanisms that control histone exchange are largely unknown. Here, we report a screening method for epigenetic regulators that we applied to search for histone exchange factors. The screening method is based on our finding that global chromatin changes in mutant cells can be inferred from chromatin states on short DNA barcodes. By analyzing the chromatin status of DNA barcodes of many yeast mutants in parallel, we identified positive and negative regulators of histone exchange. In particular, we find that the HAT-B complex promotes histone turnover. HAT-B is known to acetylate the tails of newly synthesized histones, but its role in chromatin assembly has been unclear. Hif1, the nuclear binding partner of HAT-B in the NuB4 complex, also promotes histone exchange but by non-overlapping mechanisms. These results provide a new perspective on pathways of histone exchange.

## Introduction

The epigenetic landscape in the cell is dynamic and shaped by histone modifying and demodifying enzymes. In addition, histones themselves can also be dynamic; they can be moved along the DNA through the action of ATP-dependent nucleosome remodeling enzymes or can be evicted and replaced by new histones. Many histone modifying and remodeling enzymes have been identified and several factors have been found to be involved in changing nucleosome occupancy during gene activation and repression [1]–[3]. Recent studies indicate that histones can also be replaced by replication-independent mechanisms that do not involve obvious changes in nucleosome occupancy [3]–[9]. The replacement of existing chromatin-bound histones by newly synthesized histones most likely affects the stability of chromatin marks and thereby epigenetic mechanisms of gene regulation.

Histone replacement or turnover requires assembly and disassembly of nucleosomes, processes that most likely involve the action of histone chaperones. Chaperones are acidic proteins that bind the highly basic soluble histone proteins and thereby prevent non-specific interactions of histones with other proteins and DNA [10]–[12]. The HAT-B complex is one of the factors that binds newly synthesized histones H3 and H4 in the cytoplasm [13]. This evolutionary conserved complex, composed of the chaperone Hat2 and the acetyltransferase Hat1 (also known as Kat1), acetylates newly synthesized soluble histone H4 on lysine 12 (H4K12) and lysine 5 (H4K5) [14]–[17]. Hat1 specifically acts on soluble histones because it is inactive towards chromatin-bound nucleosomal histones [13]. Hat1 is the founding (and still only known) member of the family of type B HATs, which are cytoplasmic and specific for free histones [13], [14]. Whether the HAT-B complex or its acetyltransferase activity towards the H4 tail has a role in subsequent steps of histone trafficking or chromatin assembly is not well understood [14]. Cells lacking the HAT-B complex show no growth defect, indicating that acetylation of newly synthesized histones by Hat1 is not essential for replication-dependent histone deposition [14]. In addition, the acetylation marks introduced by HAT-B are removed upon deposition of new histones in chromatin [14]. However, several studies have indicated connections between Hat1 and chromatin [15], [18]–[24]. In addition, recent biochemical studies suggest that HAT-B guides newly synthesized histones from the cytoplasm to the nucleus, where it binds to the histone chaperone Hif1 to form the NuB4 complex and hand over the histones to other chaperones such as Asf1 [25], [26]. Asf1 is involved in the stimulation of H3K56 acetylation on soluble histones prior to their deposition [11], [12]. By binding to the chromatin assembly factor complex (CAF1) and chaperone Rtt106, Asf1 can subsequently deliver histones for deposition at the replication fork [27]–[30]. In addition, Asf1 can bind to the HIR complex and thereby deliver histones for replication-independent histone deposition [11], [12], [27]–[29], [31], [32]. How chaperones affect histone assembly and disassembly is still largely unknown but recent studies are starting to reveal some of the underlying mechanisms [30], [33]–[36].

We recently developed Recombination-Induced Tag Exchange (RITE) as an assay to measure histone turnover under physiological conditions [7]. RITE is a genetic pulse-chase method in which replacement of old by new histones can be examined by immunoblots or chromatin immunoprecipitation (ChIP). To unravel the significance of the high rate of histone turnover that we and others observed in yeast [4]–[9], [37], the underlying mechanisms will need to be identified. However, identification of genes involved in histone turnover is not straightforward. Screening for mutants that affect epigenetic processes is usually carried out using indirect read-outs such as activity of reporter genes or developmental phenotypes. Mutants that affect histone post-translational modifications have also been identified by global proteome analysis [38]. However, it is not clear whether and how histone turnover affects gene expression, reporter genes, or developmental phenotypes. As a consequence, no indirect reporter assays are available to screen for histone turnover genes by mutant hunts. The alternative, direct assessment of chromatin changes in mutant clones is typically laborious (involving ChIP-sequencing or ChIP-on-chip) and is usually not suitable for genetic screening. To speed up the discovery of histone turnover pathways, we directly interrogated chromatin structure using RITE combined with methods that have been developed for parallel analysis of fitness phenotypes in yeast [39], [40]. Using this strategy we identified mutants that either positively or negatively affected histone turnover and we provide the first *in vivo* evidence for a function of the NuB4 complex in histone exchange.

## Results

### Outline of a barcode screen for histone turnover mutants

**Figure 1:**
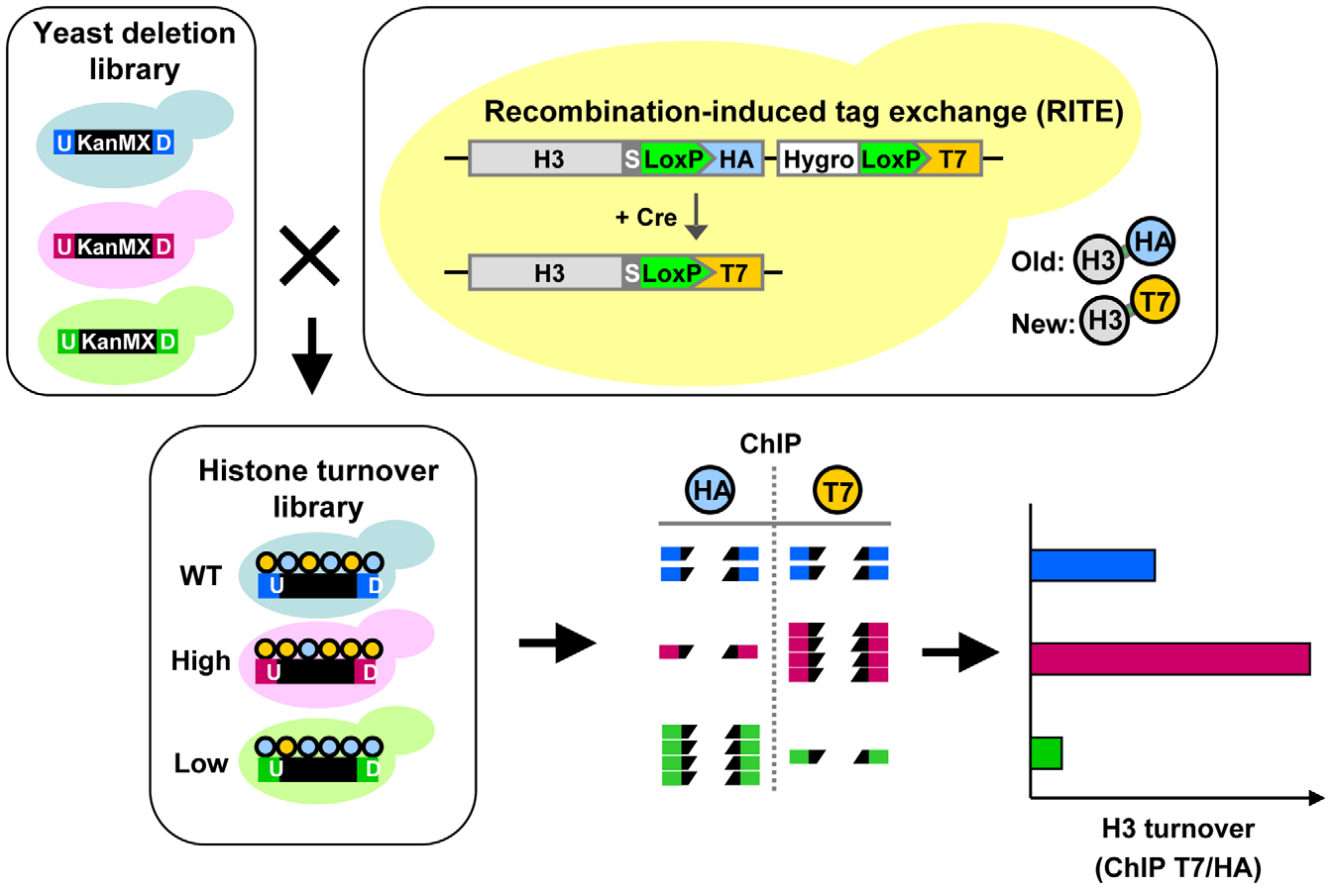
Combining Epi-ID with RITE to screen for histone turnover mutants. Each mutant in the yeast deletion library contains at the location of the deleted gene a common selectable marker gene (KanMX; black box) flanked by two unique barcodes: UpTag and DownTag (U/D). A set of deletion mutants was crossed with an H3-HAT7 RITE strain to switch epitope tags on histone H3 and monitor replacement of old by new histones in mutants (histone turnover library). Following a RITE assay and ChIP (HA and T7) on a pool of mutants, barcode abundance in each ChIP experiment was measured by deep sequencing. After normalizing the datasets, histone turnover at each barcode was calculated by taking the ratio of new/old (T7/HA) histone ChIP signals. Predicted results of mutants with higher and lower turnover are indicated.

The collection of gene-deletion mutants in *Saccharomyces cerevisiae* enables the systematic analysis of gene function. A pair of unique DNA barcodes (UpTag and DownTag) is present in each yeast deletion strain, flanking a common selectable marker gene used to knock out the respective genes (Figure 1). Molecular counting of the barcodes by DNA microarrays or digital counting by next-generation sequencing allows parallel analysis of the relative abundance of yeast clones in pooled cultures [40], [41]. The fitness of each yeast deletion mutant can be inferred from the changes in the relative abundance of the barcodes after exposure to the condition of interest. Using these same principles, we reasoned that in a pool of yeast deletion mutants the relative abundance of each barcode in a ChIP experiment might report on the abundance of a particular chromatin mark in that region in each mutant. Here we refer to the identification of epigenetic regulators by a barcode-ChIP-Seq approach as Epi-ID (Figure 1).

**Figure 2:**
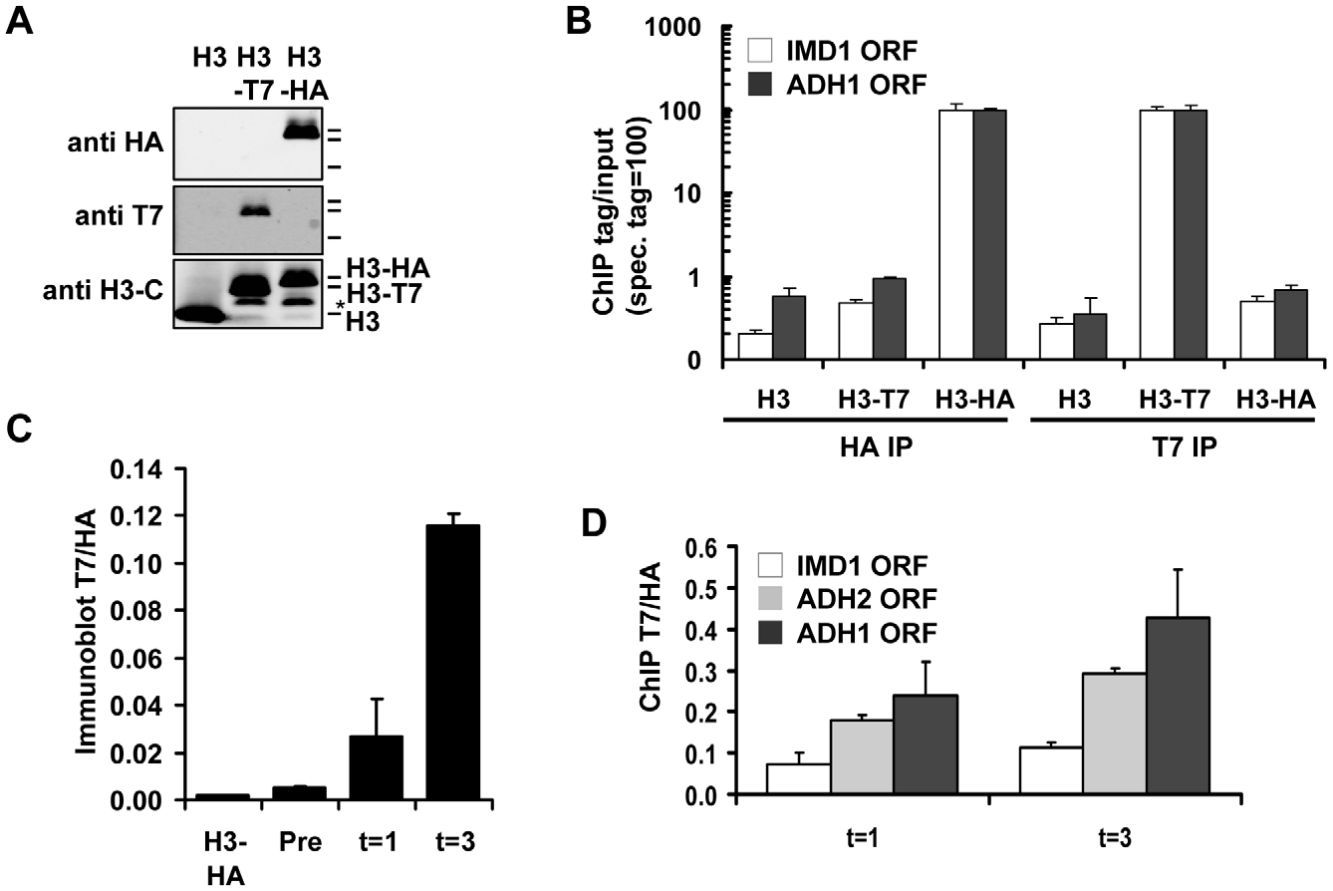
Measuring histone turnover by ChIP and immunoblot. (A) Immunoblot of strains constitutively expressing either untagged, T7- or HA-tagged H3 (strains NKI2176/NKI2300/NKI2301). Asterisk indicates an H3 degradation band. (B) ChIP of H3-HA or H3-T7 from chromatin of strains in panel A. Relative binding (binding of the antibody to chromatin with the specific tag is set to 100) is shown at two coding regions. (C) Quantification of immunoblot (Figure S2A) of whole-cell extracts of a strain constitutively expressing HA-tagged H3 or a RITE strain in which the HA switched to T7 in G0, measured before (Pre) and one and three days (t = 1 and t = 3) after induction of the switch (strains NKI2301 and NKI2215; average of two biological duplicates +/− S.E.M.). (D) ChIP of new H3-T7 over old H3-HA to determine histone turnover at three coding regions one and three days after induction of the H3-HA→T7 switch in G0 (strain NKI2215).

To explore the possibilities of Epi-ID and to search for genes involved in histone turnover we used the genetic pulse-chase method RITE to allow the detection of old and new histone H3 proteins in yeast [7] (Figure 1). Briefly, following deletion of one histone H3 gene copy, the sole remaining H3 gene was tagged with an HA tag flanked by LoxP sites, and a downstream orphan T7 tag. Initially all H3 proteins are tagged with an HA tag. Upon induction of a hormone-dependent Cre recombinase by the addition of estradiol, the HA tag in the genome is replaced by the T7 tag and from then on all newly synthesized H3 will be T7 tagged. Histone turnover results in replacement of H3-HA by H3-T7, which can be detected and quantified by immunoblot and ChIP (Figure 2). We note that histone turnover measurements obtained using RITE correlate well with measurements obtained using the previously used inducible pGAL-system to ectopically overexpress a tagged copy of histone H3 [42]. One of the advantages of RITE is that the tagged histone gene is expressed from its endogenous promoter, and old and newly synthesized histone H3 can be simultaneously detected and followed under any (physiological) condition of interest, independent of changes in nutrients to induce ectopic promoters [7], [43].

**Figure 3:**
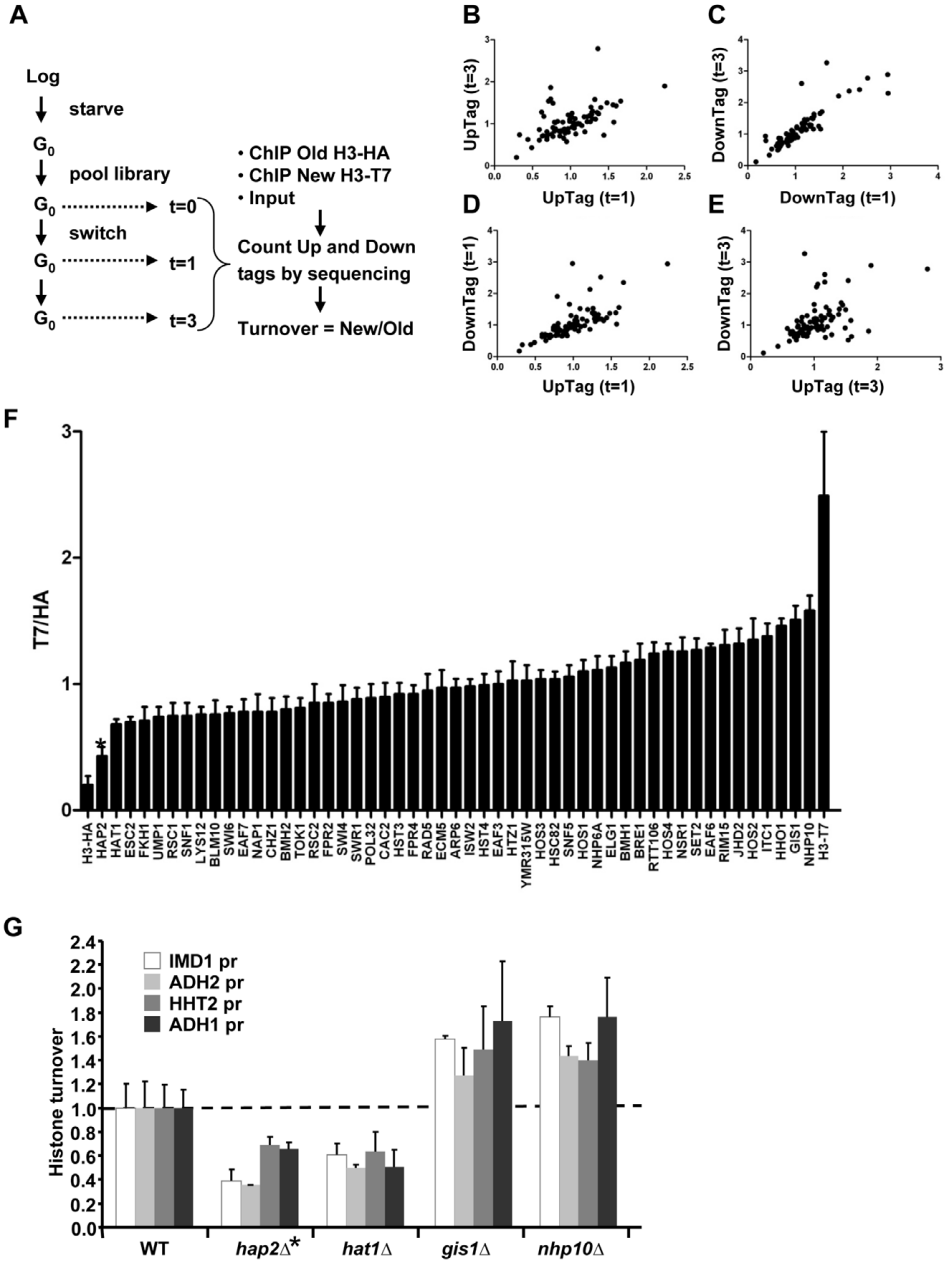
Epi-ID can identify histone turnover mutants. (A) Scheme of experimental set-up. (B–E) Comparison of new/old H3 ratios (T7/HA) of UpTags and DownTags and at two time points (one day; t = 1d, and 3 days; t = 3d), with Pearson correlations of 0.57, 0.87, 0.71, 0.52 for panels B–E, respectively. (F) Deletion mutants with low variation in histone turnover between UpTag and DownTag barcodes and between two different time points (SD<0.17) were included for further analysis (see Materials and Methods). The T7/HA ratios of mutants are individually plotted, showing HA and T7 control strains as a reference. Error bars show variation (SD) between four samples. (G) Confirmation of two individual mutants of each of the extreme ends of the bar plot in panel F at four independent promoter regions (*IMD1*, *ADH2*, *HHT2, ADH1*) unrelated to the barcoded regions. These four loci show different transcription levels and different turnover levels in wild-type cells [7] (and see Figure S2). The mutants are derived from the histone turnover library and are isogenic to NKI4128. Turnover in the mutants (ChIP signals of T7/HA at t = 3d are plotted relative to WT for each locus (WT is set to 1). The *hap2Δ* clone (*) caused low signals due to a recombination defect and was eliminated from further analysis.

We introduced the RITE elements into 92 clones of the yeast deletion collection using Synthetic Genetic Array (SGA) analysis [44] (Figure 1). The deletions in this library represented genes known or suspected to be involved in epigenetic processes and a set of non-chromatin genes (Table S1). The clones of this new library of RITE deletion mutants were first grown separately in liquid cultures, then pooled, and subsequently arrested by starvation (Figure 3A and Figure S1). Recombination was induced to switch the epitope tags and chromatin samples were taken before and one and three days after induction of the tag switch. We previously found that yeast cells arrested by starvation (which we here refer to as G0) undergo replication-independent turnover of chromatin-bound histones [7]. In addition, we found a substantial amount of new bulk histone synthesis during three days of starvation by immunoblot analysis and ChIP (Figure 2C-2D and Figure S2). Arresting cells by starvation allows for efficient switching of the epitope tags by the induced Cre recombinase. Moreover, replication-dependent histone deposition and cell-cycle or growth rate differences between different mutants are eliminated. To measure histone turnover ChIP was performed on old (H3-HA) and new (H3-T7) histone H3. The barcode regions in the bound DNA were amplified using common primer sequences and adapters to allow parallel sequencing on the Illumina platform. Four base pair index tags were introduced in each sample to allow multiplex analysis (Figure S1). After digital barcode counting (see Materials and Methods) the relative ratio of new/old H3 was calculated as a value for replication-independent histone turnover in the pool of gene deletion mutants for each UpTag and DownTag barcode and for each of two time points after induction of the tag-switch (Figure 1, Figure 3A).

### Validation of Epi-ID and candidate mutants

We performed three analyses to test the validity of the concept of Epi-ID. First, we verified that the independent measurements of the two time points (day 1 and 3) showed similar trends (Figure 3B–3C). Second, we compared UpTags with DownTags (U and D). The overall correlation between UpTag and DownTag barcodes suggests that position effects are not a major confounder in this assay (Figure 3D–3E; but also see Discussion). The few clones that did not correlate well between different time points or between UpTag and DownTag barcodes were eliminated from further analysis (see below). Third, the barcodes of the SIR3 and SIR4 deletion mutants (which do not mate and cannot be used for genetic crosses such as SGA), were integrated in the genome of strains constitutively expressing only H3–HA or only H3-T7. These clones were combined with the RITE library pool as internal negative and positive controls, respectively. The two control strains could be separated from each other at all three time points, both at the UpTag and DownTag barcodes. They also provided an indication of the dynamic range of the turnover measurements in this assay. For further analysis, clones for which severe growth defects were observed after the tag switch (and after release of the arrest by re-feeding) were excluded to eliminate mutants in which the new H3-T7 tagged histone may not be fully functional or causes tag-specific rather than true turnover effects (see Materials and Methods). Only those clones were included that showed low variation between the two time points and between UpTag and DownTag. The two control strains are shown as a reference (Figure 3F).

Of the resulting set of deletion mutants that passed the selection criteria, two clones with the lowest and two clones with the highest turnover signal were selected to examine whether the mutants affected turnover at loci independent of the barcode sequences. Each clone was grown individually and arrested by starvation. After induction of the epitope tag switch histone turnover was examined by ChIP-qPCR at four independent loci unrelated to the barcoded region analyzed in the parallel screen (promoter regions of *IMD1*, *ADH2*, *HHT2*, and *ADH1*) (Figure 3G). The changes in histone turnover at these four loci was similar to the changes measured at the barcodes, confirming that the chromatin changes of the barcodes reflected overall changes in the genome (Figure 3G). Nhp10 and Gis1 were found to be negative regulators of histone turnover. Hat1 positively regulated histone turnover. For every turnover experiment, the efficiency of the tag switch (percent of cells that had undergone a Cre-mediated recombination event) was determined (Table S2). By a colony plating assay we noticed that cells lacking *HAP2* showed very poor Cre-mediated recombination, which was most likely the cause of the low ratio of new/old H3 in this clone (Figure S3). This clone was excluded from further analysis. Given the high conservation of Hat1 and its known activity towards new histones, we focused our further studies on Hat1.

### The role of Hat1 in histone turnover

**Figure 4:**
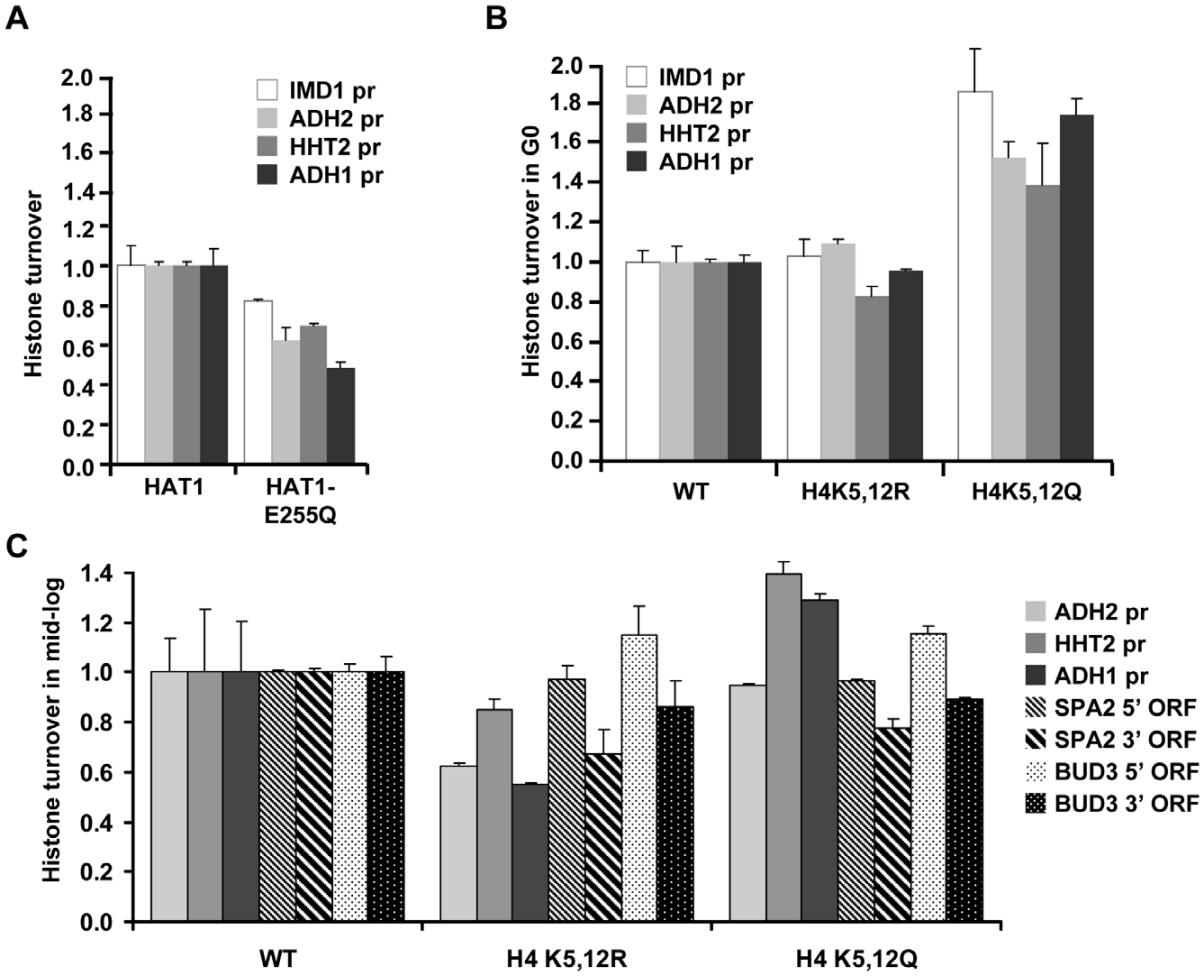
Role of Hat1 activity and localization in histone turnover. (A) Histone turnover (new/old H3, t = 3d G0, relative to WT) was determined in a strain expressing a catalytically compromised Hat1 protein (Hat1-E255Q; strains NKI4174/NKI4175). (B) Histone H3 turnover (new/old, t = 3d G0, relative to WT) was determined in strains expressing mutant histone H4 proteins in which lysines 5 and 12 were mutated to either arginine (H4K5/12R) or glutamine (H4K5/12Q; strains NKI2148/NKI2193/NKI2194). (C) Histone H3 turnover was determined in switched cells released from the starvation arrest by re-feeding and subsequently arrested for 5 h in G2/M as described in [7] by addition of nocodazole (new/old H3, relative to WT, in promoters and coding regions; strains as in panel B).

The histone acetyltransferase Hat1 together with the histone chaperone Hat2 forms the evolutionary conserved HAT-B complex that acetylates soluble histones. The functional consequences of Hat1's activity are not well understood. Hat1 plays a role in gene silencing [19] and DNA repair [14], suggesting that it affects chromatin structure. However, many of these phenotypes require additional mutations in the N-terminal tail of histone H3 and how chromatin is affected by Hat1 is not known. Our findings provide direct evidence that the Hat1 protein is important for efficient histone turnover *in vivo* (Figure 3F-3G). We first examined the role of Hat1's enzymatic activity. A strain containing a catalytically compromised (but not completely inactive) Hat1 protein (*HAT1-E255Q*) [19] showed a decrease in histone turnover similar to a *hat1Δ* strain (Figure 4A), suggesting that the acetyltransferase activity is important for efficient histone turnover. Hat1's primary known targets are lysines 5 and 12 of histone H4 (H4K5 and H4K12). Mutating the target lysines to arginine (H4K5,12R) did not substantially affect histone H3 turnover, whereas alanine or glutamine mutants (H4K5,12A and H4K5,12Q) showed enhanced turnover of histone H3 at most loci tested (Figure 4B and Figure S4). Arginine and lysine both contain a long hydrophobic side chain and a positive charge. Therefore, arginine might mimic the constitutively unacetylated (positively charged) state of lysine. Our results suggest that loss of the positive charge of H4K5,12 by acetylation is not sufficient to explain the role of Hat1 in histone turnover. However, changing the positively charged residues to neutral amino acids enhanced turnover. These results suggest that H4K5/K12 play a role in histone turnover but that loss of acetylation of these sites is not sufficient to cause a histone turnover defect.

Several lines of evidence suggest that H4K5 and H4K12 have evolutionary conserved roles in replication-dependent chromatin assembly [13], [26], [45]–[49] or nuclear import of histone H4 [50], [51]. However, in yeast, mutation of these lysines does not lead to growth defects and no changes in global chromatin organization have been observed [51]–[53]. To investigate the role of H4K5,12 in replicating cells we performed the tag switch in starved cells, released the switched population into fresh media, and then measured histone turnover in cells arrested in G2/M after one round of replication (monitored by FACS analysis). Cells expressing H4K5,12Q showed increased turnover at two of the three promoter regions analyzed, whereas cells expressing H4K5,12R showed decreased histone turnover (Figure 4C). In contrast, in two long coding sequences, the two H4K5,12 mutants affected turnover in a similar manner. In both H4K5,12 mutants turnover at the 3′ end was reduced relative to turnover at 5′ regions (Figure 4C). This is consistent with results we obtained previously with the H4K5,12R mutant in replicating cells and may indicate a role of these residues in 3′ to 5′ retrograde movement of old histones by passage of RNA Polymerase II [54].

### All members of the NuB4 complex promote histone turnover

**Figure 5:**
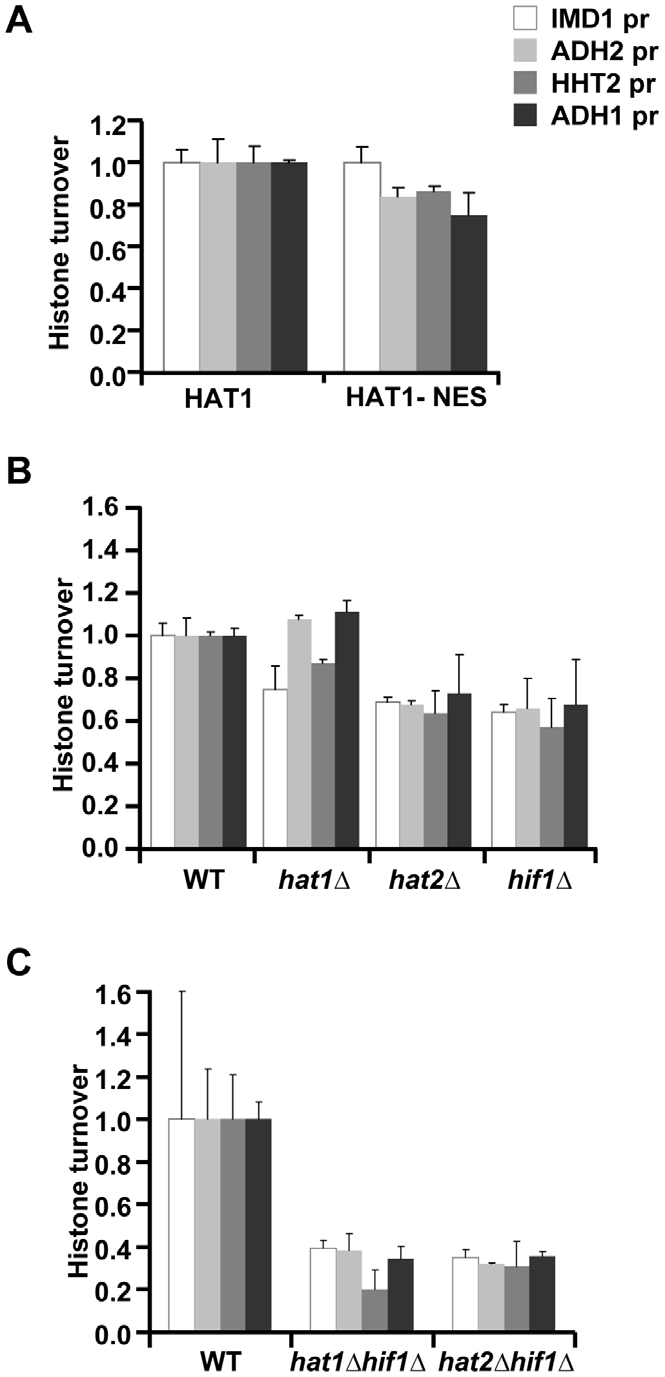
Role of the NuB4 complex in histone turnover. (A) Histone turnover (t = 3d, new/old, relative to WT) in strains in which Hat1 is predominantly maintained in the cytoplasm by fusion to a nuclear export signal (Hat1-NES). The standard error shows the spread of biological duplicates (strains NKI4176/NKI4177). (B) Histone turnover (t = 3d, new/old, relative to WT) was determined in single mutants of the three members of the NuB4 complex (strains NKI2148/NKI2191/NKI2192/NKI2187) and (C) for double mutants of *hat1Δ* and *hat2Δ* with *hif1Δ* (strains NKI4169/NKI4170). Error bars show the spread of two biological duplicates.

Hat1 in yeast and other organisms was initially identified as a cytoplasmic histone acetyltransferase [13], [14]. More recently, Hat1 was also found to be (predominantly) localized in the nucleus [14], [19], [26], [49], [55]. To investigate whether the role of Hat1 in histone turnover is mediated by a cytoplasmic or nuclear activity, we next examined the consequences of fusion of Hat1 to a nuclear export signal (Hat1-NES), which excludes Hat1 from the nucleus [19]. The NES fusion resulted in a modest decrease of histone turnover (Figure 5A), indicating that the cytoplasmic activity of Hat1 is not sufficient for Hat1's function in histone turnover and that at least part of Hat1's effect on histone turnover is mediated by a nuclear activity. To further investigate whether Hat1's role in histone turnover is indeed linked to its nuclear location, we analyzed the nuclear binding partners of Hat1. In the nucleus the members of the HAT-B complex, Hat1 and Hat2, interact with Hif1 (Hat1 Interacting Factor-1) and form the nuclear NuB4 complex [49], [55]. Hif1 belongs to the evolutionary conserved family of SHNi-TPR family of histone chaperones, which also includes Hs_NASP, Xl_N1/N2 and Sp_Sim3 [26], [56]. To examine the role of the NuB4 complex in histone turnover, we deleted Hif1 and compared this to independent deletions of Hat1 and Hat2. In this strain background, deletion of Hat1 by homologous recombination (which was confirmed by standard PCR analysis and by microarray analysis [e.g. see Figure S9]) did not affect histone turnover as much as in the mutant derived from the genetic cross with the yeast deletion collection or the catalytic mutant. The cause of this difference is unknown, but may involve differences in the genetic strain backgrounds.. However, cells lacking Hat2 or Hif1 showed reduced histone turnover, supporting the idea that the nuclear NuB4 complex plays a role in histone turnover (Figure 5B). To genetically test whether Hif1 and Hat-B affect turnover by means of a common pathway or protein complex (NuB4), we generated double mutant strains for epistasis analysis. Previous studies have shown that Hat2 is a central component of the NuB4 complex; deletion of *HAT2* disrupts the nuclear localization of Hat1 and interactions between Hif1, Hat1, and histones [19], [49], [55]. Unexpectedly, deleting either *HAT1* or *HAT2* in combination with *HIF1* resulted in a more severe decrease in histone turnover than in either one of the single mutants, suggesting that Hif1 and Hat1/Hat2 act at least in part by independent mechanisms (Figure 5C).

**Figure 6:**
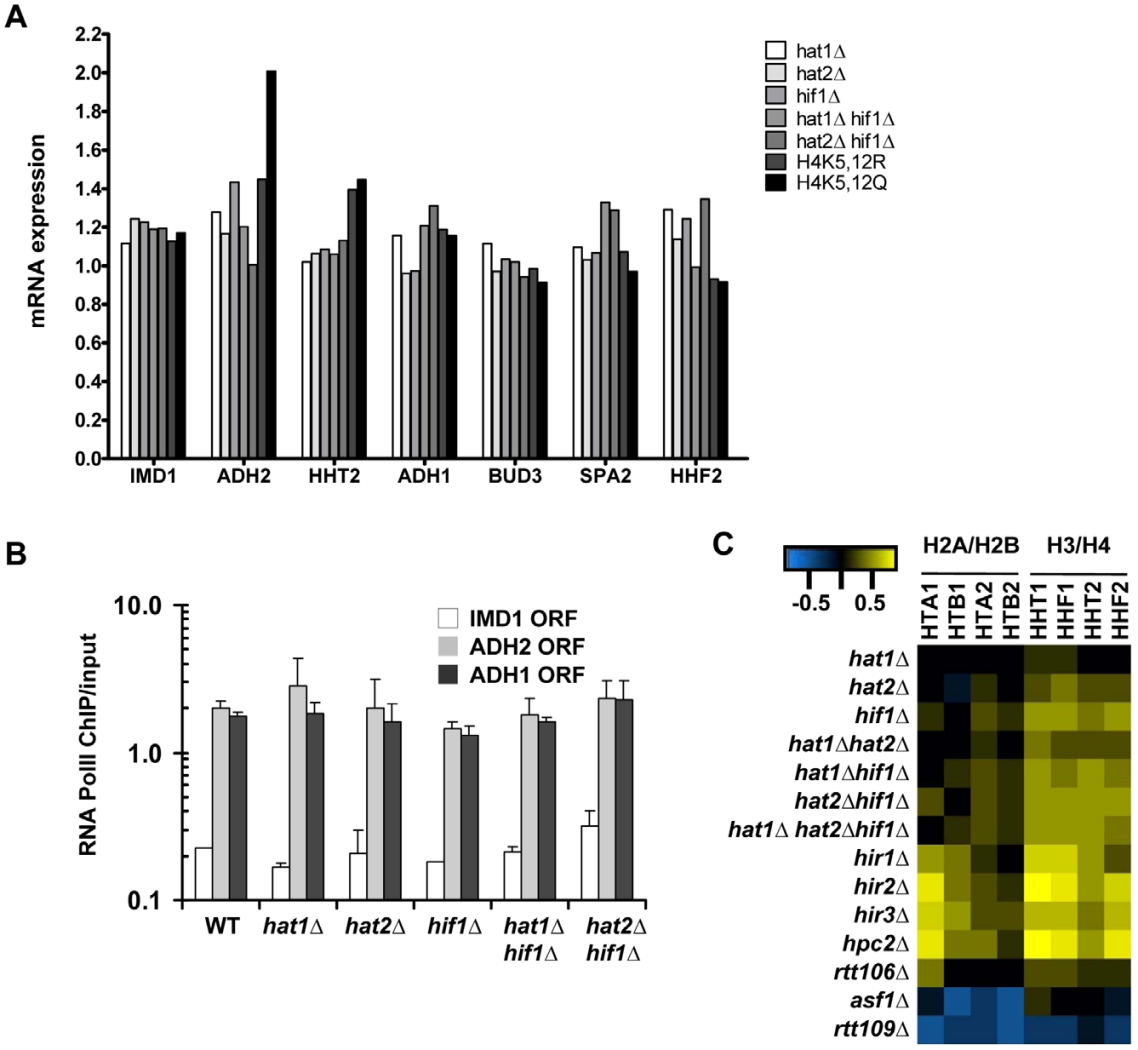
Expression changes in NuB4 mutants. (A) mRNA expression (fold change vs WT) in G0 in NuB4 and H4K5,12 mutant strains measured by microarray analysis (H3-RITE strains NKI2148/NKI2191/NKI2192/2187/NKI4169/NKI4170/NKI2193/NKI2194). (B) Transcriptional changes measured by RNA Polymerase II occupancy (ChIP) in NuB4 mutant strains (see panel A) in G0. (C) Heat map of expression changes of histone-coding genes in different histone chaperone deletion mutants (Log2) in mid-log cultures (non-RITE strains derived from BY4742). Blue indicates downregulation, yellow upregulation.

Histone turnover is strongly correlated with and induced by transcription by RNA Polymerase II [4]–[7] (and Figure S2). To investigate whether the observed G0 histone turnover defects in mutants of the NuB4 complex were caused by transcription defects we performed expression profiling and measured RNA Polymerase II occupancy. In mutant cells arrested in G0, no significant changes were found in the expression of the target genes analyzed in the turnover experiments when compared to wild-type cells (Figure 6A–6B). In addition, no significant changes (fold change >1.7, p<0.01) were found in the expression of the single H3 and H4 genes, with the exception of the H4K5,12 mutants, which showed a slight upregulation of the histone H3 gene. Thus, in G0 cells, reduced histone H3 turnover was not caused by reduced expression of (new) histones or by reduced expression of the loci at which histone turnover was measured. To compare transcriptional changes in the NuB4 mutants to other mutants, we also performed microarray analyses of NuB4 mutants made in the genetic background of the yeast deletion collection. These mutants were grown under standard mid-log conditions [57], [58]. We note that under these conditions the members of the NuB4 complex play the same role in histone turnover as in G0 (Figure S5). In general, no significant transcriptional changes were found in any of the NuB4 mutants compared to WT (fold change >1.7, p<0.01) in mid-log cultures. However, when examined in more detail, the expression profiles of mutants that contain a deletion of *HIF1* and to a lesser extent *HAT2*, showed upregulation of the genes encoding histone H3 and H4 in mid-log cultures (Figure 6C). Regulation of histone gene expression seems to be a common property of nucleosome assembly factors [27], [59], providing further support for a link between the NuB4 complex and histone turnover.

**Figure 7:**
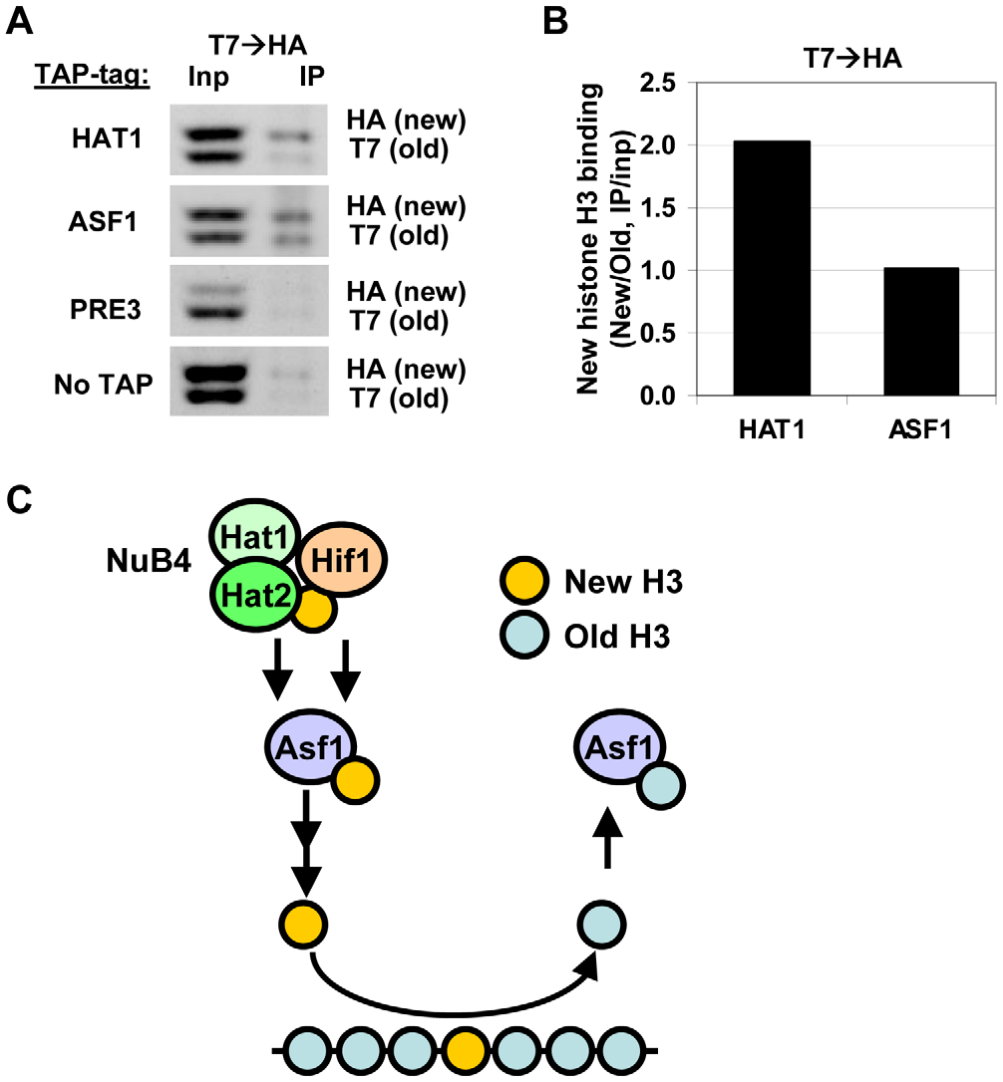
Hat1 and Asf1 bind a different subset of the soluble histone pool. (A) Following a RITE epitope-tag switch (H3-T7H3-HA) in cells arrested by starvation, cells were released in fresh media and harvested four hours later. From these cells, expressing a mix of old (T7) and new (HA) histone H3 proteins, TAP-tagged Hat1 and Asf1 were immunoprecipitated. Bound histone proteins were analyzed by immunoblots against the C-terminus of histone H3. H3-HA and H3-T7 are separated due to a size difference. Tap-tagged Pre3, a proteasome core subunit, and no-TAP strain were used as a negative control (strains NKI4187/NKI4192/NKI4195/NKI4179). (B) Signals were quantified using an Odyssey imaging system. H3 binding efficiencies were calculated by determining the IP signal relative to the input signal, after subtraction of the background signal determined by the Pre3 and NoTap controls. Strains with swapped tags (H3-HA→H3-T7) showed a similar result (Figure S6). (C) Model for pathways of histone turnover. Hat1 predominantly binds new histone H3 (yellow), whereas Asf1 binds to new as well as old (blue) histones. Hif1 and Hat1/Hat2 have non overlapping functions suggesting that they do not solely act via the NuB4 complex. Previous biochemical studies [25], [26] showed that the NuB4 members bind to Asf1 and may transfer new histones to this chaperone for subsequent nucleosome assembly.

Biochemical studies suggest that the NuB4 complex interacts with Asf1, which led to the suggestion that NuB4 might hand over newly synthesized histones to Asf1 for subsequent transfer to nucleosome assembly factors [25], [26], [60]. However, the histone genes clearly respond differently to deletion of *ASF1* than to deletion of genes encoding members of the NuB4 complex [27], [59] (Figure 6C), suggesting a more complex relationship. Unfortunately, we could not test the genetic relationship between Asf1 and Hat1 because deletion of Asf1 in the strain background used for the RITE assay is lethal, similar to what has been reported previously [61]. To investigate the connection between Hat1 and Asf1 by alternative means, we used RITE as a genetic pulse-chase tool to examine the nature of the histone molecules bound to each protein. Rather than indirectly inferring the origin of the histones (new or chromatin derived) from the pattern of post-translational modifications, the epitope tag-switch pulse-chase allows for a direct distinction between resident and newly synthesized histones. In cells that had recently undergone a tag switch on H3 and therefore contained a mix of new and old histone H3, affinity purified Hat1 bound both new and old histones with a preference for new histones (Figure 7A–7B and Figure S6). Asf1 also bound both new and old histones but without a preference for new histones. (Figure 7A–7B and Figure S6). The binding of Hat1 and Asf1 to a different subset of the pool of soluble histones suggests that they affect different steps of chromatin assembly and disassembly.

## Discussion

Using RITE as a biochemical-genetic pulse-chase tool, we previously observed rapid exchange of histone H3 in chromatin in yeast cells outside S-phase [7]. Similar results have been reported using an inducible pGAL-system to overexpress an ectopic tagged histone H3 copy [4]–[6], [37], [62], [63]. By using RITE, in contrast to the pGAL system, the tagged old and new histone H3 species are expressed by the endogenous H3 promoter from the endogenous chromosomal location [43]. Therefore, the high levels of histone exchange observed with RITE were not caused by misregulation of histone H3 expression. Indeed, qRT-PCR and microarray analyses showed that RITE strains containing tagged H3 express very similar H3 mRNA levels as wild-type cells containing untagged H3 at different phases of the cell cycle [7] (Figure S7). Interestingly, although histone mRNAs are cell cycle regulated and peak in S-phase when the demand for new histones is highest [64], [65], histone H3 mRNA expression is still relatively high outside S-phase, providing an explanation for the abundant synthesis of new histones outside S-phase [7]. To investigate the biological function of histone turnover and its consequences for chromatin structure and function, we developed the Epi-ID barcode screen for chromatin regulators and combined it with RITE. In this screen we found that Hat1 and subsequently also the other members of the NuB4 complex positively regulate histone turnover. To our knowledge, our data provide the first evidence that a Type B histone acetyltransferase complex regulates histone assembly *in vivo.* Hat1 was the first histone acetyltransferase identified [13], [66]. It is part of a multi-subunit complex that interacts with histone chaperones and acetylates free histones but is inactive towards nucleosomal histones [14], [26]. The biological significance of these biochemical activities of the Hat1 complexes remained elusive [14] although in genetic tests Hat1 was found to play a role in gene silencing and DNA damage response [14]. However, manifestation of these phenotypes required additional mutations in the N-terminal tail of histone H3 and whether these chromatin-related phenotypes are related to histone deposition defects remained unknown.

The known and conserved substrates of HAT-B/NuB4 are lysines 5 and 12 of histone H4 [14]. Mutation of these residues has revealed functions in histone H4 nuclear import and chromatin assembly [48], [50], [51]. However, H4K5,12 mutants generally show no major growth phenotypes or global changes in chromatin organization [24], [48], [52], [53]. Here we found a positive effect of H4K5,12A and H4K5,12Q mutants on histone turnover in promoters, suggesting that NuB4 may exert its turnover function via H4K5/K12 acetylation. However, H4K5,12R, mimicking the hypo-acetylated state of these lysines, did not cause a turnover defect (Figure 4). One possible explanation of these results is that NuB4 has additional substrates that contribute to its role in histone turnover [67]. We do not know whether other substrate lysines on histones or perhaps non-histone proteins are also involved and play roles redundant with the acetylated histone H4 tail.

The nuclear function for HAT-B in histone turnover (Figure 5) indicates that HAT-B's role in histone metabolism may be more complex than previously anticipated and extends beyond the acetylation of newly synthesized histones. This is in line with observations that Hat1 can be recruited to chromatin at origins of replication and DNA double strand breaks [20], [21] and with the role of members of the NuB4 complex in depositing histones following repair of a DNA double strand break [18]. Unexpectedly, our studies revealed that Hat1 and Hat2 act in parallel with Hif1, and that Hat1 and Asf1 bind a different subset of the soluble histone pool. In previous studies Hat1/Hat2, Hif1, and Asf1 have been shown to bind to each other [26], which led to the suggestion that Asf1 acts downstream of Hat1/Hat2/Hif1 and passes on new histones acetylated on H4K5/K12 (and H3K56) to chromatin assembly factors CAF-I, HIR, and Rtt106 [11], [12]. Our results suggest that Hat1/Hat2, Hif1 and Asf1 act, at least in part, via distinct pathways of chromatin assembly and/or disassembly (Figure 6C and Figure 7). The equal binding of Asf1 to new and old histones suggests that Asf1 may be involved in depositing as well as escorting histones evicted from chromatin (Figure 7C), which is in concordance with the finding that H3K56 acetylation (mediated by Rtt109/Asf1) is a mark of new histones, yet is important for histone eviction and nucleosome destabilization [11], [33]. Indeed, histone chaperones may not exclusively function in chromatin assembly [68]. For example Nap1, which can escort H3/H4 and H2A/H2B and assemble histone octamers into nucleosomes, but may orchestrate this by promoting nucleosome disassembly [33]. Another example is CAF1, which is involved in replication-coupled assembly of new histones into chromatin, yet histone H3 bound to this complex (or to Rtt106 or Asf1) contains methylated H3K79 [30], which is a mark of chromatin-bound histones [69], [70].

What are the functional consequences of altering histone turnover? Histone turnover might affect several aspects of the epigenome, such as nucleosome occupancy, DNA accessibility, or dynamics of histone modifications. No changes in growth or cell cycle progression were observed for single, double, or triple *hat1Δ*, *hat2Δ*, *hif1Δ* mutants (Figure S8) and no significant transcriptional changes were observed (see Figure 6 and Material and Methods). Apparently, slowing down turnover of histone H3 by loss of the NuB4 complex has no profound consequences under these conditions. Deletion of *HAT2* or *HIF1* resulted in a moderate increase in expression of the genes encoding histone H3 and H4 in mid-log cultures (Figure 6C). We expect that this may be a response to the histone turnover defects caused by deletion of Hat2 and Hif1, since deletion of Hat1, which overall has a lower impact on histone turnover, did not affect histone gene expression. It is possible that the phenotypes of the *hat1Δ* strain are relatively weak because of compensation of Hat1's activity by other HATs, such as Gcn5, which acetylates newly synthesized histone H3 [71]. In our microarray analyses, we did not observe significant upregulation of mRNA levels of other (putative) HATs in G0 cultures (Figure S9). Overall, our results indicate that loss of NuB4 function alone has no major consequences for global chromatin organization.

What is the function of histone turnover? Histone turnover leads to turnover of histone modifications and can thereby affect the pattern as well as dynamics of the epigenome. When a chromatin state is controlled by two opposing activities (e.g. modification and demodification by turnover) this could lead to a more rapid establishment a new equilibrium after perturbation of the epigenome, such as during DNA replication or after exposure to stress (e.g. see [72]). Based on models proposed for histone acetylation one could also envision that dynamic turnover (cycles of modification and demodification) rather than the steady state may be relevant for chromatin function [73]. Alternatively, histone turnover could counteract the accumulation of histone modifications that are less susceptible to demodification. For example, methylation of histone H3K79, which accumulates in a non-processive manner on aging histones [72] is enriched in genomic regions that show low histone turnover and retain old histone H3 molecules, suggesting that histone inheritance and dynamics help shape the epigenome [54]. The identification of additional mutants in future screens will help to further deconstruct the pathways of histone turnover and to discover their biological significance.

In the Epi-ID screen we also identified Gis1 and Nhp10 as negative regulators of histone turnover. Gis1 is a zinc-finger transcription factor involved in regulation of stress genes [74] and contains a Jumonji domain, which has been associated with histone demethylase activity [75]. Gis1 has also been reported to bind to several factors involved in DNA metabolism [76]. It will be interesting to test whether any of these Gis1-binding proteins or its putative demethylase activity is involved in this novel function of Gis1. Nhp10 is a non-essential subunit of the essential INO80 chromatin remodeling complex that can move or mobilize nucleosomes. Two recent studies suggest a role for INO80 in redeposition of histones during induced transcription [77], [78]. That Nhp10 slows down histone turnover provides further support for the idea that the INO80 complex can help to preserve the chromatin architecture during transcription. In an Epi-ID screen using 1536 chromosome biology mutants in which the old and new tags on histone H3 were swapped (old-T7 and new-HA), *NHP10* and *GIS1* mutants also showed more histone turnover (data not shown), indicating that the phenotypes observed were not caused by tag-specific effects and that Epi-ID can be scaled up.

The application of Epi-ID is not restricted to histone turnover. In fact, in screens for other epigenetic marks such as histone modifications or nucleosome occupancy Epi-ID, can be applied without the elaborate genetic crosses and genetic switches that are required for screens based on the RITE pulse chase assay. Future applications in yeast may benefit from other barcoded mutant collections that are being developed [79]–[81]. Although our study suggests that position effects of the barcoded marker are not major confounders in Epi-ID and can be (at least in part) excluded by comparing UpTag with DownTag barcodes, DNA barcodes at a common genetic locus separated from the gene deletion would be preferable for epigenetic screens. The recently developed Yeast Barcoders Library represents such a collection in which barcoded markers are integrated at the common HO locus thereby providing opportunities to further expand and improve the application of Epi-ID in yeast [82]. Finally, the basic principles of this approach should also be applicable to barcoded mutant libraries in other organisms, such as barcoded episomes, or transposon or virus insertion libraries.

## Materials and Methods

### Yeast strains, plasmids, and media

Yeast strains used in this study are listed in Table S3. Yeast media were described previously [7]. The pilot set of mutants (see Table S1) was manually made from the *MAT*a haploid gene knockout library (Open Biosystems). H3-RITE (strain NKI4114) was crossed in duplicate with 92 mutants by Synthetic Genetic Array analysis [44] with the following modifications. After mating, diploids were selected and kept on Hygromycin, G418 and CloNat triple selection on rich media for one night. After 13 days on sporulation media a series of selections followed to select for the proper MAT haploids: twice on haploid MATa selection (YC-His+Can+SAEC), twice on triple resistance selection (YC-His+Can+SAEC+MSG+ Hygromycin, G418 and CloNat), and then twice on YC-His-Leu to select for H3-RITE strains in which the second, untagged, copy of H3 was deleted by insertion of LEU2. NKI2178 and NKI4179 are derivatives of BY4733. Plasmid pTW087, which was used to make strain NKI2178, was made by inserting a 6xHis tag behind the HA tag into pFvL118 [7] by PCR mutagenesis. Plasmid pTW088, which was used to make strain NKI4197, was made by replacing the HA tag in pTW081 [7] by a HA-6xHIS tag generated by PCR amplification from pTW087. NKI4128 was derived from a cross between Y7092 and NKI4004 [7], [44]. NKI8013 and NKI4140 were derived from NKI4179 and NKI4128 after elimination of the first tag and HphMX marker by induction of recombination. *BAR1* was deleted using pMPY-ZAP. NKI2176 was derived from BY4733 using reagents described previously [7]. NKI2215 and NKI2216 were derived from NKI2176 by targeting the RITE cassettes from pFvL118 and pTW081 to the HHT2 locus [7]. NKI2300 and NKI2301 were derived from NKI2215 and NKI2216, respectively, after elimination of the first tag and HphMX marker by induction of recombination.

### Switch assay and follow-up

The tag switch assay was performed as described previously [7] with a few adjustments. Briefly, all strains were grown in 600 µl YPD containing Hygromycin (200 µg/ml, Invitrogen) in 96-well format for three nights at 30°C. Cells were then pooled in 50 ml of saturated media without Hygromycin containing 1 µM β-estradiol (E-8875, Sigma-Aldrich). Approximately 1x10^9^ cells were fixed with 1% formaldehyde for 15 minutes before addition of β-estradiol (t = 0), after 16 hours (t = 1) and after 3 additional days (t = 3) for chromatin immunoprecipitation. In the follow-up analysis of candidate turnover mutants, we identified several possible confounders in our specific turnover screen. In certain mutants low turnover measurements were caused by lack of the Cre-recombinase mediated tag-switch. These mutants were excluded from the follow-up studies. Some mutants showed severe loss of viability after the tag-switch and when released into fresh media. These clones were also excluded from the follow-up studies to avoid clones in which the new H3-T7 tagged histone may not be fully functional and possibly causes tag-specific rather than a physiological turnover effects.

### ChIP-Seq

ChIP was performed as described previously [7]. One tenth of each sample was taken as input. After DNA isolation all samples were amplified using different SeqiXU1 primers in combination with P7U2 for the amplification of the UpTag and SeqiXD1 primers with P7D2 for amplification of the DownTag (primers are listed in Table S4). PCR amplification was conducted in 50 µl reactions using Phusion DNA polymerase (Finnzymes) with the following conditions: 10 cycles of 98°C/15 s, 56°C/15 s, 72°C/20 s; 20 cycles of 98°C/15 s, 72°C/15 s, 72°C/20 s. The amplicons of different conditions were pooled per tag, size separated on a 2% gel and the correct sized amplicons were excised and extracted using a Qiagen gel purification column. In a subsequent PCR reaction equal amounts of DNA of the UpTag and DownTag were amplified with primers P5seq and either P7U2 or P7D2 to attach the adapter fragments necessary for cluster formation and sequencing on the Illumina genome analyzer. PCR amplification was conducted in 50 µl reactions using Phusion® DNA polymerase with the following conditions: 10 cycles of 98°C/15 s, 56°C/15 s, 72°C/20 s; 20 cycles of 98°C/15 s, 72°C/25 s. The indexed barcode libraries were analyzed on an Illumina GAII genome analyzer and processed as described below.

### Mapping sequence reads

The indexed barcode libraries were analyzed on an Illumina GAII. Sequence reads were expected to have the following composition: 4 bp index (i), 18 bp common UpTag (U1) or 17 bp common DownTag (D1) primer sequence, up to 20 bp unique UpTag or DownTag barcode sequence. A database of expected sequence reads was generated by combining the barcode sequences originally designed (http://www-sequence.stanford.edu/group/yeast_deletion_project/deletions3.html) with corrected sequences based on re-sequencing of the barcodes of the yeast diploid heterozygous deletion collection [40], [83]. Multiplex indexed barcodes were identified at position 1 to 6 allowing no mismatches. Barcodes were identified starting at position 22, 21, or 23, respectively, initially allowing no mismatches over a length of 11 nt. Unidentified reads were further analyzed in a second round by FASTA using the optimal alignment of gene tags. FASTA alignments were only considered with a minimal alignment length of 10 bases and a minimal identity of 90%. Only alignments that start within 2 bases from position 22 were allowed and alignments were not allowed to stop more than 5 bases from the end of the barcode. A set of unused barcodes [39], [41] was used to verify that allowing mismatches did not lead to a high false discovery rate and to determine cut-offs for P-values (see below). Out of a total number of 7446311 reads, 6249225 could be assigned to an indexed barcode amplicon. The mapped sequence reads were binned in UpTag and DownTag barcode fractions, further binned in sample fractions using the 4 bp indexes, and then the relative abundance of each barcode within each specific bin was determined using reads per million counts for each bin. Based on the behavior of the unused barcodes, to avoid false positive assignments clones with outlying up / down ratio counts (P-value <0.01) in any of the indexed samples were excluded from further analysis. Histone turnover was determined by calculating the ratio of T7 ChIP over HA ChIP for t = 1 and t = 3 days (t = 1, t = 3) and for the UpTag and DownTag barcodes. Only clones with a low variation between these four samples (SD <0.17; and thereby only clones for which both the UpTag and DownTag barcode were identified) were included for further analysis. Cut offs for variation were set such that all false positive identifications of the unused barcode set were excluded. Of the 92 clones in the screen, 53 were included in the final dataset. Drop-outs were caused by the genetic crossing or by the stringent selection criteria.

### Follow-up analysis of individual mutants

Strains were grown individually to saturation in 50 ml of YPD; ChIP was performed only on samples after three days of saturation. ChIP DNA was quantified in real-time PCR using the SYBR Green PCR Master Mix (Applied Biosystems) and the ABI PRISM 7500 as described previously. An input sample was used to make a standard curve, which was then used to calculate the IP samples, all performed in the 7500 fast system software. As a measurement for turnover, the amount DNA of the T7-IP was divided over the HA-IP. The antibodies used for ChIP and immunoblots are HA (12CA5), T7 (A190-117A, Bethyl or 69522-3, Novagen), H3 C-terminus [7], RITE-spacer+LoxP [7], RNA PolII/Rpb1 (8WG16). Primers used for qPCR are listed in Table S5.

### TAP-IP

The equivalent of 1x10^9^ cells was washed with cold TBS, resuspended in 1ml cold TBS with a protease inhibitor cocktail. All steps were performed cold at 4°C unless otherwise stated. Cells were briefly spun and the pellet was frozen at −80°C. The pellet was dissolved in 400 µl lysis buffer (25 mM Hepes pH 7.9, 50 mM NaCl, 0.1% NP-40, 1 mM EDTA, 10% glycerol) containing a protease inhibitor cocktail. Cells were lysed by the addition of 400 µl glass beads and vortexing for 15 min on a multivortex. The total lysate was spun at maximum speed for 5 min, the soluble fraction was transferred to a new tube and 1 ml of lysis buffer was added. The lysate was then spun for 5 min 14K, transferred to a new tube, then spun for 15 min 14K and again transferred to a new tube. Of this fraction 50 µl was used as input, the rest was incubated with 30 µl IgG beads (Invitrogen) for 2 hrs. The beads were washed three times with cold lysis buffer for 5 min and once with TEV buffer (50 mM Tris pH 8, 0.5 mM EDTA, 50 mM NaCl, and 1 mM DTT). The beads were resuspended in 100 µl TEV buffer to which 175 µg recombinant TEV protease is added and kept overnight. The soluble fraction contains the immunoprecipitated fraction and was analyzed by quantitative immunoblotting. Lysates were separated on a 16% polyacrylamide gel and blotted onto 0.45 µm nitrocellulose membrane. Membranes were blocked with 2% Nutrilon (Nutricia) in PBS. Primary antibody incubations were performed overnight in Tris-buffered saline-Tween with 2% Nutrilon, anti-HA (mouse 12CA5), anti-T7 (Novagen, 1∶1000) or a polyclonal antibody obtained against the LoxP peptide (1∶2500) [7]. Secondary antibody incubations were performed for 45 minutes using LI-COR Odyssey IRDye 800CW (1∶12.000). Immunoblots were subsequently scanned on a LI-COR Odyssey IR Imager (Biosciences) using the 800 channel. Signal intensities were determined using Odyssey LI-COR software version 3.0.

### FACS analysis

To monitor cell cycle progression and cell cycle arrests the DNA content of the cells was measured by flow cytometry as described previously [7], using SYTOX Green and a 530/30 filter (Becton-Dickinson). Analysis was performed using FCSexpress2.

### Expression profiling

Each mutant strain was profiled four times from two independently inoculated cultures and harvested in early mid-log phase in synthetic complete medium with 2% glucose or harvested in starvation conditions in rich media as described above for the turnover experiments. Sets of mutants were grown alongside corresponding WT cultures and processed in parallel. Dual-channel 70-mer oligonucleotide arrays were employed with a common reference WT RNA. All steps after RNA isolation were automated using robotic liquid handlers. These procedures were first optimized for accuracy (correct FC) and precision (reproducible result), using spiked-in RNA for calibration [84]. After quality control, normalization, and dye-bias correction [85], statistical analysis for mid-log cultures was performed for each mutant versus the collection of 200 WT cultures as described by Lenstra et al [57]. The reported FC is an average of the four replicate mutant profiles versus the average of all WTs. HAT1, HAT2, and HIF1 single, double, and triple mutants in the BY4742 background were not different from wild type (less than three genes changed p<0.01, FC >1.7 after removal of WT variable genes). Mutants in G0 were compared to replicates of the corresponding wild-type RITE strain. Due to variability under conditions of starvation [86] we did not perform genome-wide statistical analyses of expression changes in G0 cultures.

### Accession numbers

Microarray data have been deposited in ArrayExpress under accession numbers E-TABM-1175 (mutants) and E-TABM-773/E-TABM-984 (200 WT replicates), as well as in GEO under accession number GSE30168.

## Supporting Information

Figure S1 Scheme showing PCR amplification strategy of barcoded regions around the KANMX selectable marker gene. A first round of amplification introduces an index sequence to barcoded regions of each experimental condition. A second round of amplification introduces the sequences required for Illumina sequencing. All mutants were grown individually to starvation, and then pooled into one culture. Before induction and one day and three days after induction of the tag switch samples were taken for HA and T7 immunoprecipitation and input. Each of these conditions was assigned a 4 bp index sequence as listed.
(TIF)

Figure S2 Immunoblot and ChIP analysis of new histone H3-T7 in starved cells. (A) Immunoblot analysis of new histone H3-T7 and old histone H3-HA before and after induction of the tag switch in starved cells. The H3-HAT7 switch (strain NKI2215) was performed in duplicate. Quantification is shown in Figure 2C. (B) The amount of new H3-T7/input was determined for three loci at two time points after induction of the tag switch in starvation (strain NKI2215). Cells containing 100% T7 or 100% HA show IP efficiencies of approximately 2.5–10% (data not shown).
(TIF)

Figure S3 Recombination defect in *hap2Δ* mutant. Upon deletion of *HAP2*, the efficiency of recombination (percent of cells that had lost the Hygromycin resistance gene) was impaired, leading to more background recombination before and less recombination after induction of the switch.
(TIF)

Figure S4 Role of H4K5 and K12 in histone turnover. The amount of histone turnover at the promoter region of four genes was determined by dividing the ChIP signal of H3-T7 over H3-HA (new/old) and plotted relative to WT. The standard error shows the spread of biological duplicates. Histone turnover was measured in histone H4 mutants carrying mutated lysines 5 and 12 to alanines (H4K5/12A; strains from Figure 4B and NKI2195). Data for H4K5/12R and H4K5/12Q mutants are duplicated from Figure 4B for comparison.
(TIF)

Figure S5 Role of NuB4 in histone turnover in replicating cells. Histone turnover (ChIP new/old H3) was determined in replicating cells by inducing the tag switch in cells that had been growing in log phase for at least 16 hours and by taking samples two population doublings after induction of Cre recombinase. During this time-period the population of cells is undergoing the Cre-mediated recombination event in an asynchronous manner (see Table S2). Wild type is set to 1, turnover was determined at four promoter regions (strains NKI2148/NKI2191/NKI2192/2187).
(TIF)

Figure S6 Old and new histone H3 binding to Hat1 and Asf1. (A) As explained in Figure 7, following a RITE epitope-tag switch (H3-HAH3-T7 and H3-T7H3-HA) tap-tagged Hat1 and Asf1 were immunoprecipitated from cells expressing a mix of old and new histone H3 proteins. Bound histone proteins were analyzed by immunoblots against the C-terminus of histone H3. H3-HA and H3-T7 are separated due to a size difference (strains NKI4174/NKI4191/NKI4195/NKI2178). (B) Signals were quantified using an Odyssey imaging system. H3 binding efficiencies were calculated by determining the IP signal relative to the input signal, after subtraction of the background signal determined by the Pre3 and NoTap controls.
(TIF)

Figure S7 Effects of histone H3 tags on mRNA expression levels. Microarray analysis of mRNA expression of target genes in RITE strains in mid-log expressing 100% HA-tagged histone H3 or 100% T7-tagged histone H3 (changes vs isogenic RITE strain expressing untagged H3; strains NKI2176/NKI2300/NKI2301). HHT2 and HHF2 represent the genes encoding histone H3 and H4, respectively.
(TIF)

Figure S8 Growth of mutants of the NuB4 complex. (A) Wild-type (BY4742) and NuB4 mutant strains (all derived from BY4742) were grown under the conditions indicated after spotting on agar plates in 10-fold dilution series. Photos were taken after incubating the plates for 2–3 days. (B) Analysis of cell cycle profiles by staining for DNA content and analysis by flow cytometry. Strains were grown at 30°C in YPD media and harvested in log phase.
(TIF)

Figure S9 Expression of genes encoding HATs in NuB4 mutants. Microarray analysis of mRNA expression changes in genes encoding (putative) HATs in NuB4 and H4K5,12 mutant strains (fold change vs isogenic wild-type RITE strain in G0 t = 3d). Strains: NKI2148/NKI2191/NKI2192/NKI2187/NKI4169/NKI4170/NKI2193/NKI2194.
(TIF)

Table S1 Yeast strains in Epi-ID histone turnover screen.
(DOC)

Table S2 Recombination efficiencies in tag switch experiments.
(DOC)

Table S3 Yeast strains.
(DOC)

Table S4 Primers used for deep sequencing.
(DOC)

Table S5 qPCR primers.
(DOC)
