# Supplementary figures and images for: A Barcode Screen for Epigenetic Regulators Reveals a Role for the NuB4/HAT-B Histone Acetyltransferase Complex in Histone Turnover
Source: PLoS Genet. 2011 Oct 6;7(10):e1002284. doi: 10.1371/journal.pgen.1002284 (PMC3188528; doi:10.1371/journal.pgen.1002284)

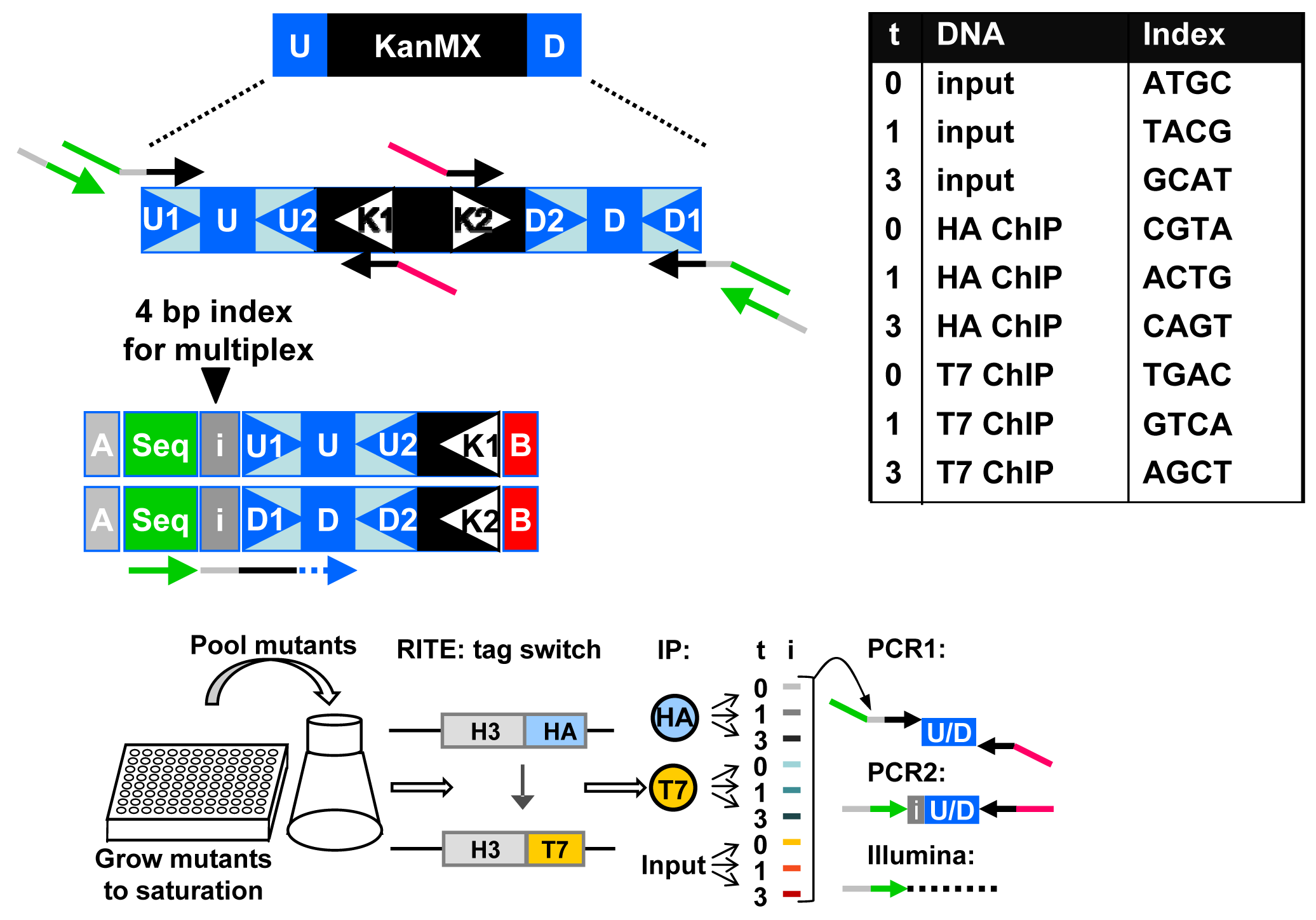

Supplement: Figure S1 — Scheme showing PCR amplification strategy of barcoded regions around the KANMX selectable marker gene. A first round of amplification introduces an index sequence to barcoded regions of each experimental condition. A second round of amplification introduces the sequences required for Illumina sequencing. All mutants were grown individually to starvation, and then pooled into one culture. Before induction and one day and three days after induction of the tag switch samples were taken for HA and T7 immunoprecipitation and input. Each of these conditions was assigned a 4 bp index sequence as listed. (TIF) [file pgen.1002284.s001.tif]

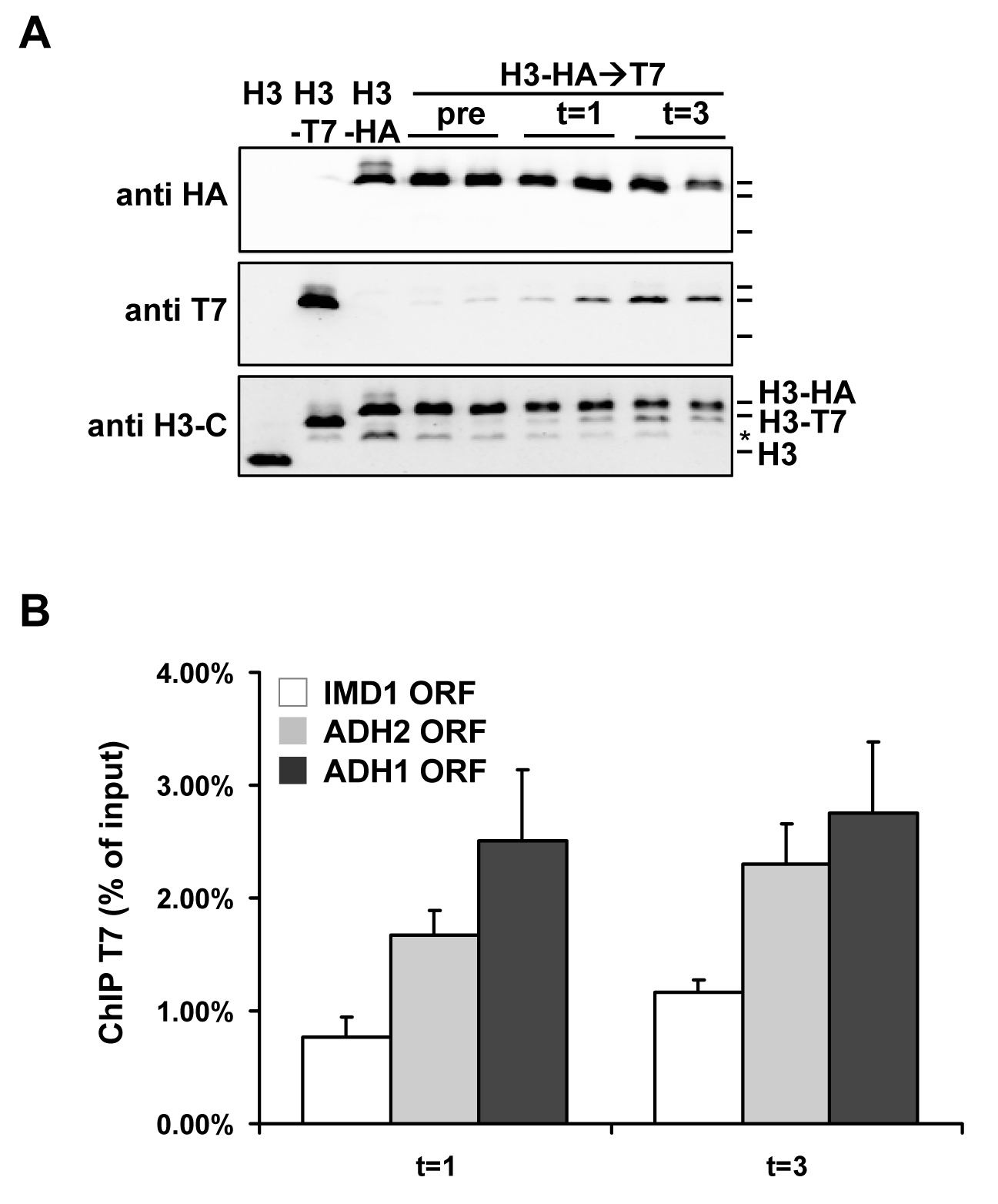

Supplement: Figure S2 — Immunoblot and ChIP analysis of new histone H3-T7 in starved cells. (A) Immunoblot analysis of new histone H3-T7 and old histone H3-HA before and after induction of the tag switch in starved cells. The H3-HAT7 switch (strain NKI2215) was performed in duplicate. Quantification is shown in Figure 2C. (B) The amount of new H3-T7/input was determined for three loci at two time points after induction of the tag switch in starvation (strain NKI2215). Cells containing 100% T7 or 100% HA show IP efficiencies of approximately 2.5–10% (data not shown). (TIF) [file pgen.1002284.s002.tif]

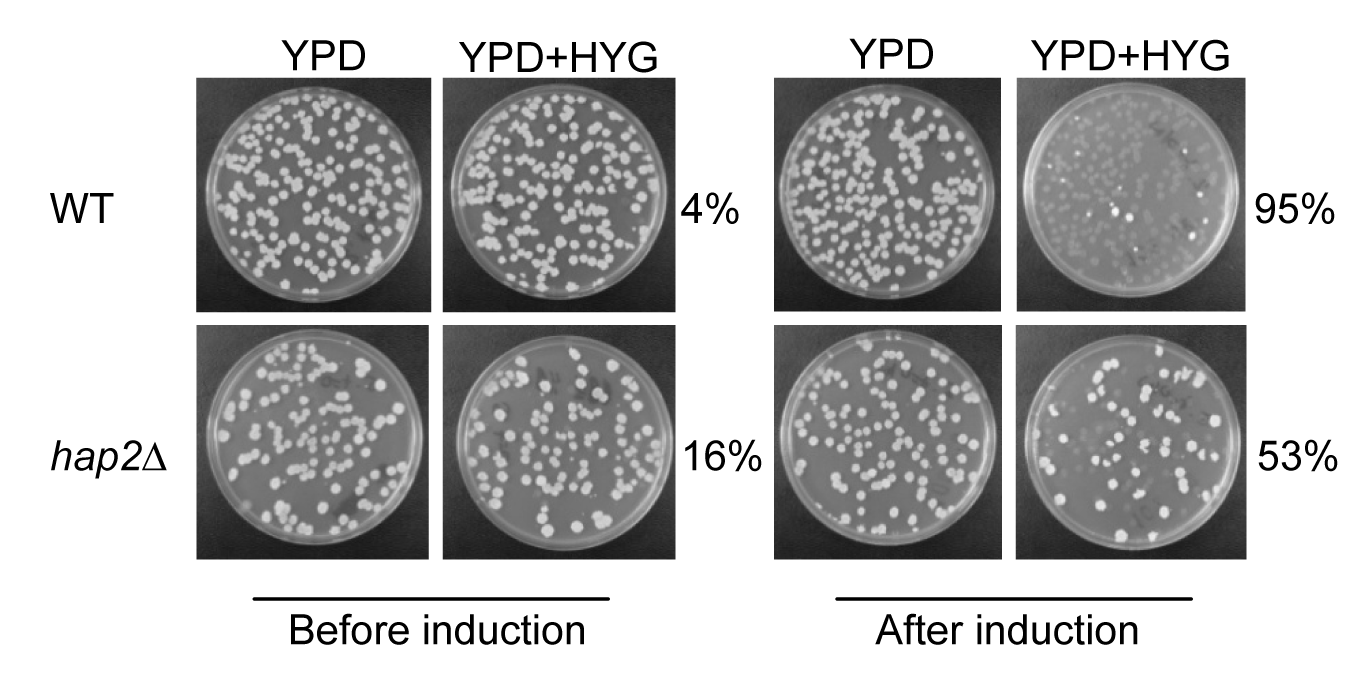

Supplement: Figure S3 — Recombination defect in hap2Δ mutant. Upon deletion of HAP2, the efficiency of recombination (percent of cells that had lost the Hygromycin resistance gene) was impaired, leading to more background recombination before and less recombination after induction of the switch. (TIF) [file pgen.1002284.s003.tif]

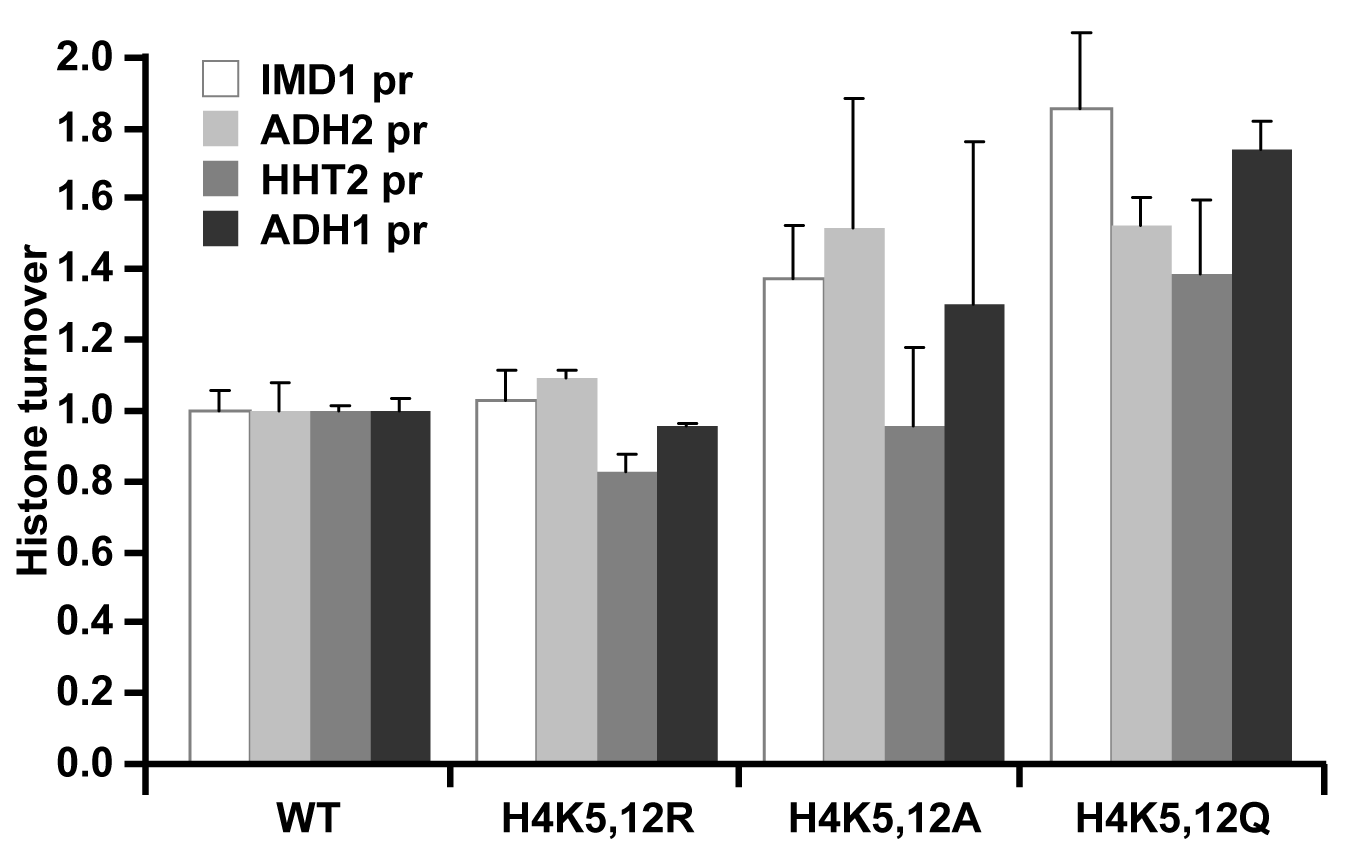

Supplement: Figure S4 — Role of H4K5 and K12 in histone turnover. The amount of histone turnover at the promoter region of four genes was determined by dividing the ChIP signal of H3-T7 over H3-HA (new/old) and plotted relative to WT. The standard error shows the spread of biological duplicates. Histone turnover was measured in histone H4 mutants carrying mutated lysines 5 and 12 to alanines (H4K5/12A; strains from Figure 4B and NKI2195). Data for H4K5/12R and H4K5/12Q mutants are duplicated from Figure 4B for comparison. (TIF) [file pgen.1002284.s004.tif]

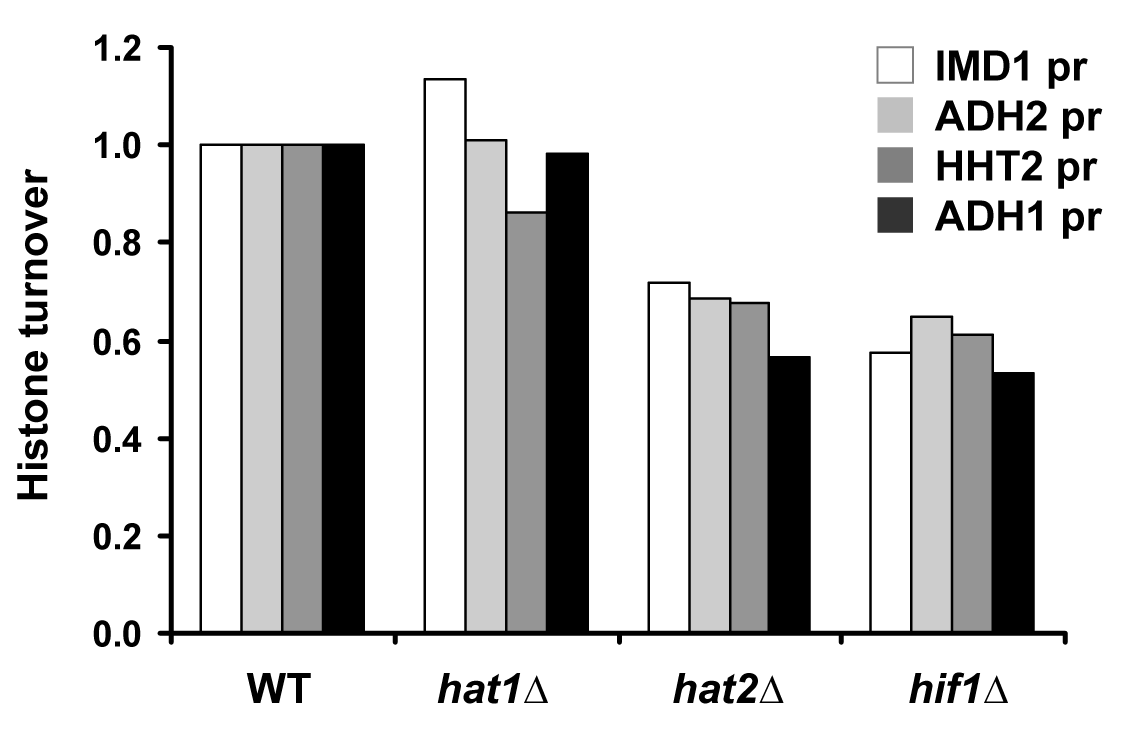

Supplement: Figure S5 — Role of NuB4 in histone turnover in replicating cells. Histone turnover (ChIP new/old H3) was determined in replicating cells by inducing the tag switch in cells that had been growing in log phase for at least 16 hours and by taking samples two population doublings after induction of Cre recombinase. During this time-period the population of cells is undergoing the Cre-mediated recombination event in an asynchronous manner (see Table S2). Wild type is set to 1, turnover was determined at four promoter regions (strains NKI2148/NKI2191/NKI2192/2187). (TIF) [file pgen.1002284.s005.tif]

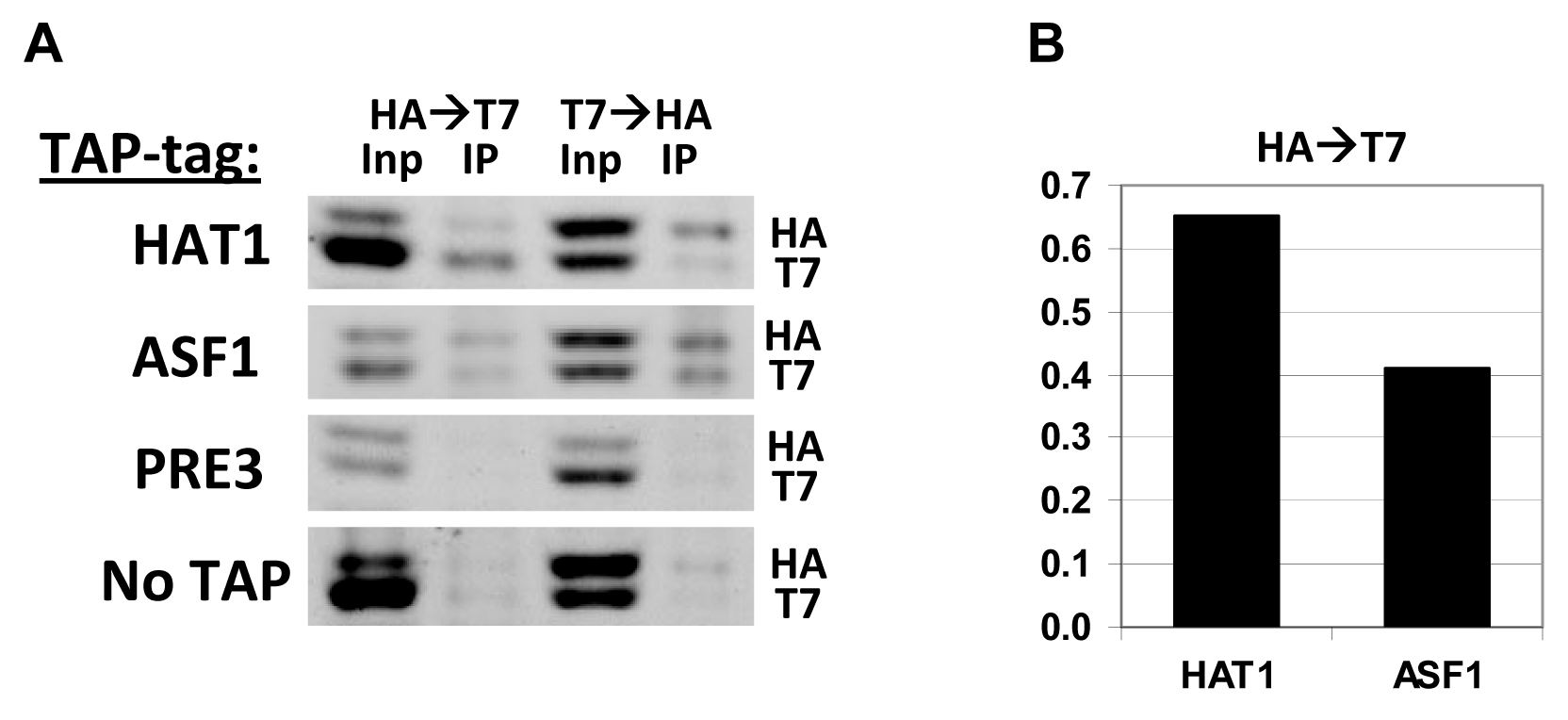

Supplement: Figure S6 — Old and new histone H3 binding to Hat1 and Asf1. (A) As explained in Figure 7, following a RITE epitope-tag switch (H3-HAH3-T7 and H3-T7H3-HA) tap-tagged Hat1 and Asf1 were immunoprecipitated from cells expressing a mix of old and new histone H3 proteins. Bound histone proteins were analyzed by immunoblots against the C-terminus of histone H3. H3-HA and H3-T7 are separated due to a size difference (strains NKI4174/NKI4191/NKI4195/NKI2178). (B) Signals were quantified using an Odyssey imaging system. H3 binding efficiencies were calculated by determining the IP signal relative to the input signal, after subtraction of the background signal determined by the Pre3 and NoTap controls. (TIF) [file pgen.1002284.s006.tif]

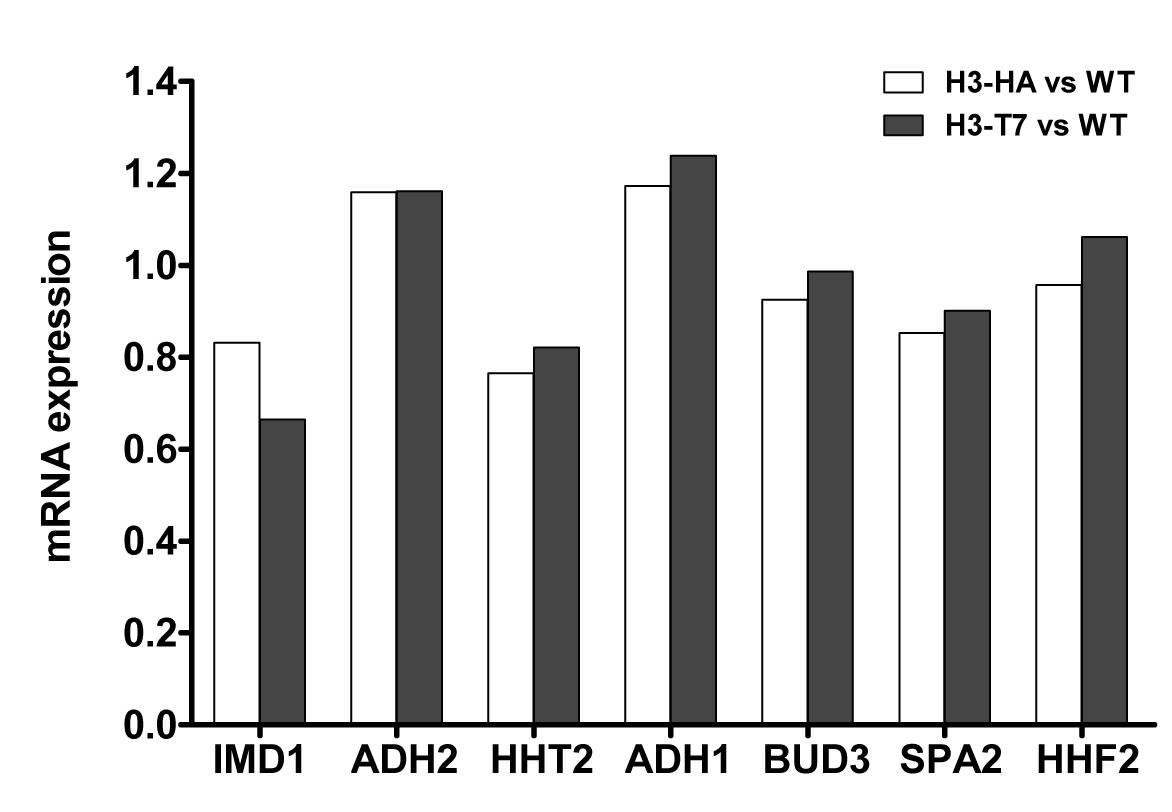

Supplement: Figure S7 — Effects of histone H3 tags on mRNA expression levels. Microarray analysis of mRNA expression of target genes in RITE strains in mid-log expressing 100% HA-tagged histone H3 or 100% T7-tagged histone H3 (changes vs isogenic RITE strain expressing untagged H3; strains NKI2176/NKI2300/NKI2301). HHT2 and HHF2 represent the genes encoding histone H3 and H4, respectively. (TIF) [file pgen.1002284.s007.tif]

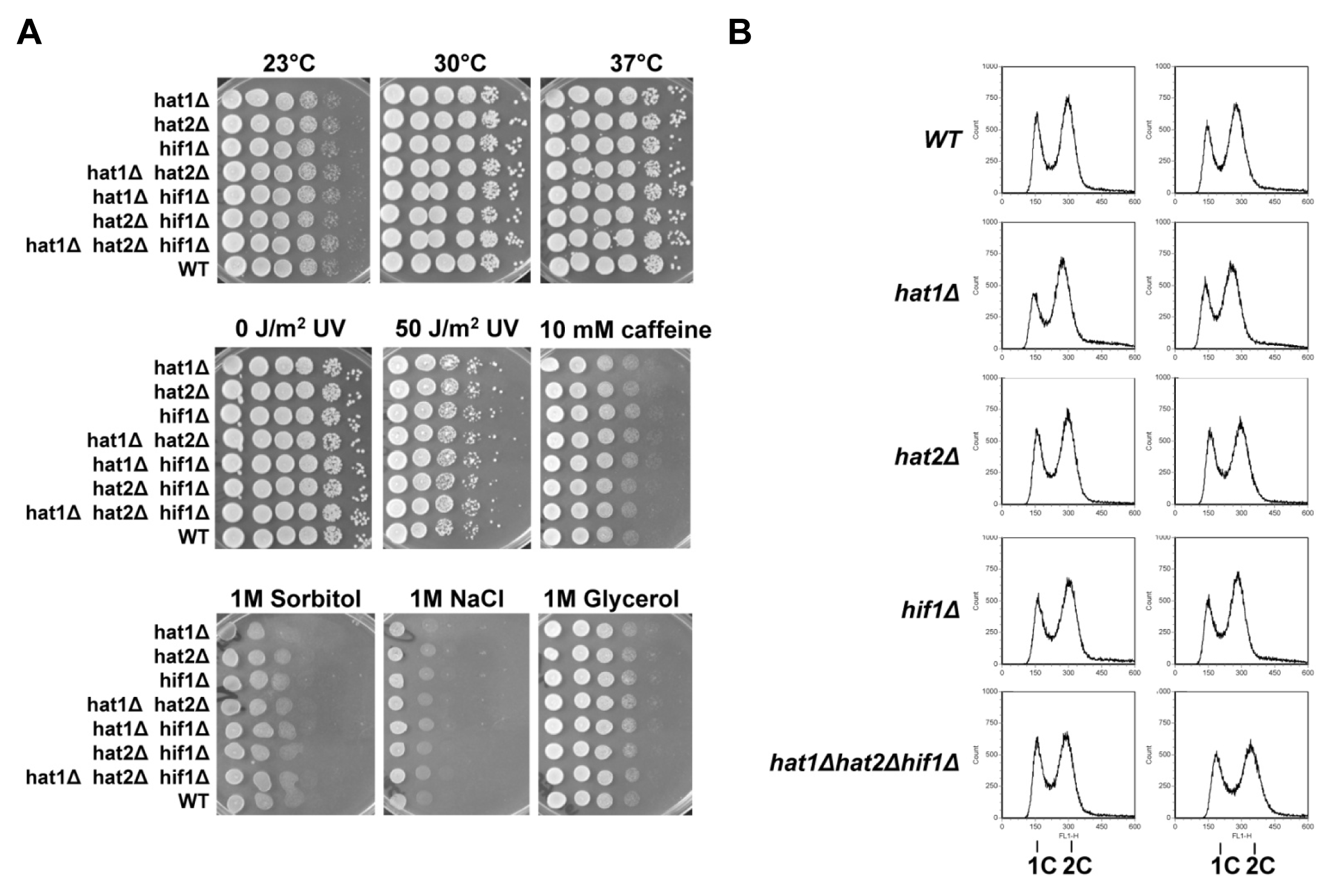

Supplement: Figure S8 — Growth of mutants of the NuB4 complex. (A) Wild-type (BY4742) and NuB4 mutant strains (all derived from BY4742) were grown under the conditions indicated after spotting on agar plates in 10-fold dilution series. Photos were taken after incubating the plates for 2–3 days. (B) Analysis of cell cycle profiles by staining for DNA content and analysis by flow cytometry. Strains were grown at 30°C in YPD media and harvested in log phase. (TIF) [file pgen.1002284.s008.tif]

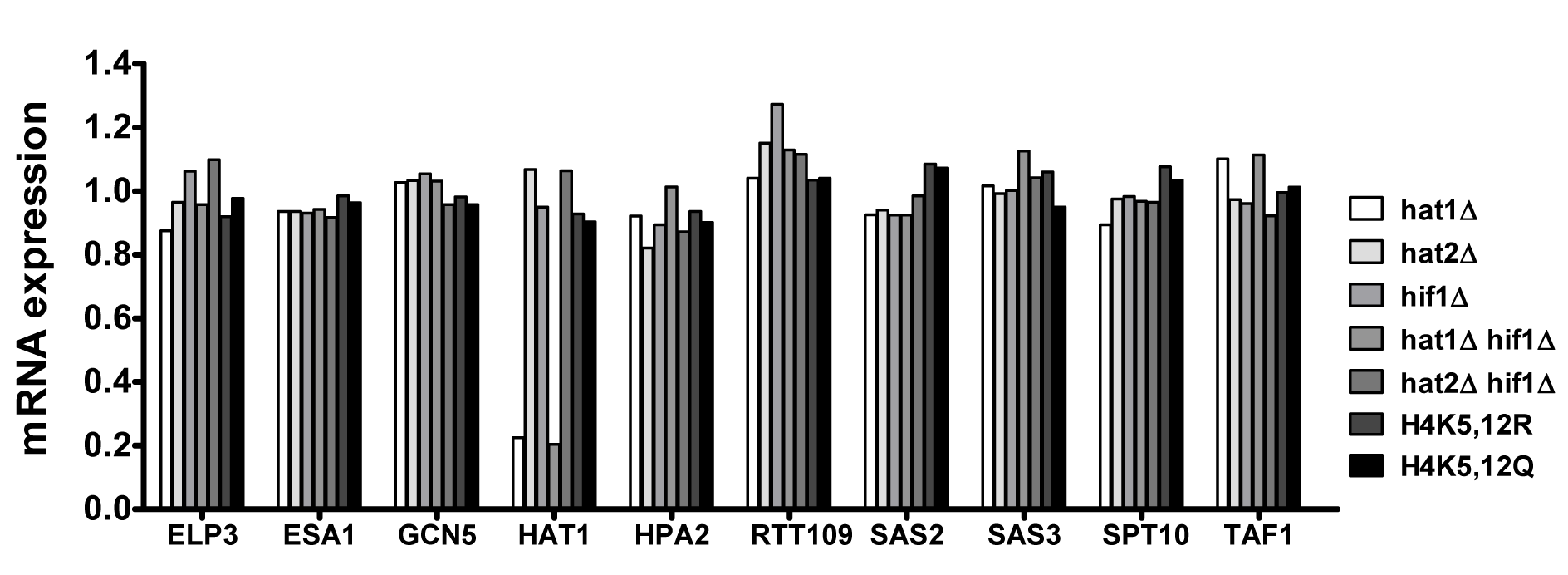

Supplement: Figure S9 — Expression of genes encoding HATs in NuB4 mutants. Microarray analysis of mRNA expression changes in genes encoding (putative) HATs in NuB4 and H4K5,12 mutant strains (fold change vs isogenic wild-type RITE strain in G0 t = 3d). Strains: NKI2148/NKI2191/NKI2192/NKI2187/NKI4169/NKI4170/NKI2193/NKI2194. (TIF) [file pgen.1002284.s009.tif]
