# Supplementary material for: A Barcode Screen for Epigenetic Regulators Reveals a Role for the NuB4/HAT-B Histone Acetyltransferase Complex in Histone Turnover
Source: PLoS Genet. 2011 Oct 6;7(10):e1002284. doi: 10.1371/journal.pgen.1002284 (PMC3188528; doi:10.1371/journal.pgen.1002284)
Supplement: Table S1 — Yeast strains in Epi-ID histone turnover screen. (DOC) [file pgen.1002284.s010.doc]

**TABLE S1: Yeast strains in Epi-ID histone turnover screen**

| ORF | Name | Included |
| --- | --- | --- |
| YAR003W | SWD1 | 0 |
| YBL008W | HIR1 | 0 |
| YBL052C | SAS3 | 0 |
| YBR114W | RAD16 | 0 |
| YBR173C | UMP1 | 1 |
| YBR175W | SWD3 | 0 |
| YBR195C | MSI1 | 0 |
| YBR245C | ISW1 | 0 |
| YBR289W | SNF5 | 1 |
| YDL002C | NHP10 | 1 |
| YDL074C | BRE1 | 1 |
| YDR096W | GIS1 | 1 |
| YDR099W | BMH2 | 1 |
| YDR143C | SAN1 | 0 |
| YDR191W | HST4 | 1 |
| YDR216W | ADR1 | 0 |
| YDR227W | SIR4-T7 | 1 |
| YDR334W | SWR1 | 1 |
| YDR363W | ESC2 | 1 |
| YDR392W | SPT3 | 0 |
| YDR477W | SNF1 | 1 |
| YDR519W | FPR2 | 1 |
| YER030W | CHZ1 | 1 |
| YER051W | JHD1 | 0 |
| YER111C | SWI4 | 1 |
| YER164W | CHD1 | 0 |
| YER169W | RPH1 | 0 |
| YER177W | BMH1 | 1 |
| YFL007W | BLM10 | 1 |
| YFL013C | IES1 | 0 |
| YFL033C | RIM15 | 1 |
| YGL058W | RAD6 | 0 |
| YGL115W | SNF4 | 0 |
| YGL133W | ITC1 | 1 |
| YGL163C | RAD54 | 0 |
| YGL194C | HOS2 | 1 |
| YGL237C | HAP2 | 1 |
| YGL244W | RTF1 | 0 |
| YGR056W | RSC1 | 1 |
| YGR159C | NSR1 | 1 |
| YIL094C | LYS12 | 1 |
| YIL112W | HOS4 | 1 |
| YIL131C | FKH1 | 1 |
| YJL093C | TOK1 | 1 |
| YJL168C | SET2 | 1 |
| YJL176C | SWI3 | 0 |
| YJR043C | POL32 | 1 |
| YJR082C | EAF6 | 1 |
| YJR119C | JHD2 | 1 |
| YJR140C | HIR3 | 0 |
| YKL113C | RAD27 | 0 |
| YKR029C | SET3 | 0 |
| YKR048C | NAP1 | 1 |
| YLR032W | RAD5 | 1 |
| YLR085C | ARP6 | 1 |
| YLR182W | SWI6 | 1 |
| YLR357W | RSC2 | 1 |
| YLR442C | SIR3-HA | 1 |
| YLR449W | FPR4 | 1 |
| YML074C | FPR3 | 0 |
| YML102W | CAC2 | 1 |
| YMR127C | SAS2 | 0 |
| YMR176W | ECM5 | 1 |
| YMR186W | HSC82 | 1 |
| YMR223W | UBP8 | 0 |
| YMR315W | YMR315W | 1 |
| YNL021W | HDA1 | 0 |
| YNL135C | FPR1 | 0 |
| YNL136W | EAF7 | 1 |
| YNL206C | RTT106 | 1 |
| YNL334C | SNO2 | 0 |
| YOL012C | HTZ1 | 1 |
| YOL068C | HST1 | 0 |
| YOR025W | HST3 | 1 |
| YOR038C | HIR2 | 0 |
| YOR080W | DIA2 | 0 |
| YOR123C | LEO1 | 0 |
| YOR141C | ARP8 | 0 |
| YOR144C | ELG1 | 1 |
| YOR290C | SNF2 | 0 |
| YOR304W | ISW2 | 1 |
| YPL001W | HAT1 | 1 |
| YPL086C | ELP3 | 0 |
| YPL116W | HOS3 | 1 |
| YPL127C | HHO1 | 1 |
| YPL240C | HSP82 | 0 |
| YPL254W | HFI1 | 0 |
| YPR018W | RLF2 | 0 |
| YPR023C | EAF3 | 1 |
| YPR052C | NHP6A | 1 |
| YPR068C | HOS1 | 1 |
| YPR193C | HPA2 | 0 |
| Total included | | 53 |
| Total in starting set | | 92 |
