# Supplementary material for: A Barcode Screen for Epigenetic Regulators Reveals a Role for the NuB4/HAT-B Histone Acetyltransferase Complex in Histone Turnover
Source: PLoS Genet. 2011 Oct 6;7(10):e1002284. doi: 10.1371/journal.pgen.1002284 (PMC3188528; doi:10.1371/journal.pgen.1002284)
Supplement: Table S2 — Recombination efficiencies in tag switch experiments. (DOC) [file pgen.1002284.s011.doc]

**TABLE S2: Recombination levels in RITE tag switch experiments**

| **Recombination levels** | | **Pre induction** | | **Post induction** | |
| --- | --- | --- | --- | --- | --- |
| **Figure** | **Strain** | **clone 1** | **clone 2** | **clone 1** | **clone 2** |
| 2C-D,S2 | WT | 6 | 2 | 98 | 99 |
| 3G | WT | ≤ 1 | 4 | 96 | 97 |
|  | WT | 8 | 4 | 95 | 94 |
|  | hap2Δ | 16 |  | 53 |  |
|  | hat1Δ | 1 | 1 | ≥ 99 | 94 |
|  | gis1Δ | 1 | 4 | 90 | 83 |
|  | nhp10Δ | 8 | 7 | 95 | 97 |
| 4A | WT HAT1-TAP | nd | nd | ≥ 99 | ≥ 99 |
|  | HAT1-E255Q-TAP | nd | nd | ≥ 99 | ≥ 99 |
| 4B | WT | 2 | 1 | 97 | 97 |
|  | WT | ≤ 1 |  | 98 |  |
|  | H4K5,12R | 3 | 3 | 87 | 98 |
|  | H4K5,12Q | ≤ 1 | 1 | 92 | 97 |
| 4C | WT | ≤ 5 | ≤ 5 | 91.0 | 94.0 |
|  | H4 K5,12R | ≤ 5 | ≤ 5 | 68.0 | 83.0 |
|  | H4 K5,12Q | ≤ 5 | ≤ 5 | 96.0 | ≥ 99 |
| S4 | H4K5,12A | 2 | 2 | 99 | 87 |
| 5A | WT HAT1-Myc | nd | nd | ≥ 99 | ≥ 99 |
|  | HAT1-NES-Myc | nd | nd | ≥ 99 | ≥ 99 |
| 5B | WT | 2 | 1 | 97 | 97 |
|  | hat1Δ | 3 | 2 | 95 | 97 |
|  | hat2Δ | 4 | 1 | 93 | 97 |
|  | hif1Δ | 3 | 2 | 95 | 99 |
| 5C | WT | 6 | 10 | 97 | 97 |
|  | WT | ≤ 1 |  | 98 |  |
|  | hat1Δ hif1Δ | 6 | 7 | 71 | 96 |
|  | hat2Δ hif1Δ | ≤ 1 | 7 | 91 | 96 |
|  | | | | | |
| S5 |  |  | | **Post induction** | |
|  | **Strain** | **Pre induction** | | **2h** | **4h** |
|  | WT | ≤ 1 | | 48.0 | 73.0 |
|  | hat1Δ | ≤ 1 | | 51.0 | 70.0 |
|  | hat2Δ | ≤ 1 | | 41.0 | 66.0 |
|  | hif1Δ | ≤ 1 | | 44.0 | 74.0 |
