# Supplementary material for: A Barcode Screen for Epigenetic Regulators Reveals a Role for the NuB4/HAT-B Histone Acetyltransferase Complex in Histone Turnover
Source: PLoS Genet. 2011 Oct 6;7(10):e1002284. doi: 10.1371/journal.pgen.1002284 (PMC3188528; doi:10.1371/journal.pgen.1002284)
Supplement: Table S3 — Yeast strains. (DOC) [file pgen.1002284.s012.doc]

**TABLE S3: Yeast strains**

| **Strain** | **Relevant genotype** | **Reference** |
| --- | --- | --- |
| NKI2148 | MATa his3Δ200 leu2Δ0 lys2Δ0 met15Δ0 ura3Δ0 hhf1-hht1∆::LEU2 hht2::HHT2-LoxP-HA-HPHMX-LoxP-T7 HIS3::P_TDH3_-CRE-EBD78 bar1∆::HisG | [1] |
| NKI4114 | MATα his3∆1 leu2∆0 ura3∆0 met15∆0 can1∆::P_STE2_-Sp-his5 hhf1-hht1∆::LEU2 hht2::HHT2-LoxP-HA-HPHMX-LoxP-T7 lyp1∆::NATMX-P_TDH3_-CRE-EBD78 | [2] |
| BY4741 | MATa his3∆1 leu2∆0 met15∆0 ura3∆0 | [3] |
| BY4733 | MATa his3∆200 leu2∆0 met15∆0 ura3∆0 trp1∆63 | [3] |
| NKI4128 | MATa his3∆1 leu2∆0 met15∆0 ura3∆0 can1∆::P_STE2_-Sp-his5 hhf1-hht1∆::LEU2 hht2::HHT2-LoxP-HA-HPHMX-LoxP-T7 lyp1∆::NATMX P_TDH3_-CRE-EBD78 | [2] |
| NKI4004 | MATa his3Δ200 leu2Δ0 lys2Δ0 met15Δ0 ura3Δ0 Δhhf1-hht1::LEU2 hht2::HHT2-LoxP-HA-HYG-LoxP-T7 | [1] |
| NKI4140 | NKI4128 after recombination HHT2-LoxP-T7 | This study |
| NKI2161 | NKI4004 DOT1::”SIR3-BC”-KANMX-DOT1 | This study |
| NKI2162 | NKI4140 DOT1::”SIR4-BC”-KANMX-DOT1 | This study |
| NKI2176 | MATa his3∆200 leu2∆0 trp1∆63 ura3∆0 met15∆0 hht1-hhf1::MET15 bar1::HisG HIS3 PTDH3_CRE_EBD78 | This study |
| NKI2215 | MATa his3∆200 leu2∆0 trp1∆63 ura3∆0 met15∆0 hht1-hhf1::MET15 bar1::HisG HIS3 PTDH3_CRE_EBD78 hht2::HHT2-LoxP-HA-HPHMX-LoxP-T7 | [2] |
| NKI2216 | MATa his3∆200 leu2∆0 trp1∆63 ura3∆0 met15∆0 hht1-hhf1::MET15 bar1::HisG HIS3 PTDH3_CRE_EBD78 hht2::HHT2-LoxP-T7-HPHMX-LoxP-HA | This study |
| NKI2300 | MATa his3∆200 leu2∆0 trp1∆63 ura3∆0 met15∆0 hht1-hhf1::MET15 bar1::HisG HIS3 PTDH3_CRE_EBD78 hht2::HHT2-LoxP-T7 | This study |
| NKI2301 | MATa his3∆200 leu2∆0 trp1∆63 ura3∆0 met15∆0 hht1-hhf1::MET15 bar1::HisG HIS3 PTDH3_CRE_EBD78 hht2::HHT2-LoxP-HA | This study |
| NKI2191 | NKI2148 hat1∆::KANMX | This study |
| NKI2192 | NKI2148 hat2∆::KANMX | This study |
| NKI2187 | NKI2148 hif1∆:: KANMX | This study |
| NKI4169 | NKI2191 hif1::NATMX | This study |
| NKI4170 | NKI2192 hif1::NATMX | This study |
| NKI2193 | NKI2148 hhf2::HHF2K5QK12Q | This study |
| NKI2194 | NKI2148 hhf2::HHF2K5RK12R | [2] |
| NKI2195 | NKI2148 hhf2::HHF2K5AK12A | This study |
| NKI2178 | MATa his3∆200 leu2∆0 trp1∆63 ura3∆0 met15∆0 hht1-hhf1::MET15 bar1::HisG HIS3 PTDH3_CRE_EBD78 hht2::HHT2-LoxP-HA-6HIS-HPHMX-LoxP-T7 | This study |
| NKI4174 | NKI2178 HAT1-TAP-KANMX | This study |
| NKI4175 | NKI2178 HAT1E255Q-TAP-KANMX | This study |
| NKI4176 | NKI2178 HAT1-MYC-TRP1 | This study |
| NKI4177 | NKI2178 HAT1-MYC-NES-TRP1 | This study |
| NKI4191 | NKI2178 ASF1-TAP-KANMX | This study |
| NKI4195 | NKI2178 PRE3-TAP-KANMX | This study |
| NKI4179 | MATa his3∆200 leu2∆0 trp1∆63 ura3∆0 met15∆0 hht1-hhf1::MET15  bar1::HisG HIS3::PTDH3_CRE_EBD78 hht2::HHT2-LoxP-T7-HPHMX-LoxP-HA-6HIS | This study |
| NKI4187 | NKI4179 HAT-TAP-KANMX | This study |
| NKI4192 | NKI4179 ASF1-TAP-KANMX | This study |
| NKI4196 | NKI4179 PRE3-TAP-KANMX | This study |
| BY4742 | MATα his3∆1 leu2∆0 ura3∆0 | [3] |
| NKI2271 | BY4742 hat1∆::NatMX | This study |
| NKI2272 | BY4742 hat2∆::NatMX | This study |
| NKI2168 | BY4742 hif1∆::KanMX | This study |
| NKI4197 | BY4742 hat1∆::KanMX hat2∆::NatMX | This study |
| NKI2269 | BY4742 hat1∆::NatMX hif1∆::KanMX | This study |
| NKI2270 | BY4742 hat2∆::NatMX hif1∆::KanMX | This study |
| NKI4198a | BY4742 hat1∆::HphMX hat2∆::NatMX hif1∆::KanMX | This study |
| NKI4198b | BY4742 hat1∆::NatMX hat2∆::HphMX hif1∆::KanMX | This study |

1. Verzijlbergen KF, Menendez-Benito V, van Welsem T, van Deventer SJ, Lindstrom DL, Ovaa H, Neefjes J, Gottschling DE, van Leeuwen F (2010) Recombination-induced tag exchange to track old and new proteins. Proc Natl Acad Sci U S A 107: 64-68.

2. Radman-Livaja M, Verzijlbergen KF, Weiner A, van Welsem T, Friedman N, Rando OJ, van Leeuwen F (2011) Patterns and mechanisms of ancestral histone protein inheritance in budding yeast. PLoS Biol 9: e1001075.

3. Brachmann CB, Davies A, Cost GJ, Caputo E, Li J, Hieter P, Boeke JD (1998) Designer deletion strains derived from Saccharomyces cerevisiae S288C: a useful set of strains and plasmids for PCR-mediated gene disruption and other applications. Yeast 14: 115-132.
