# Supplementary material for: A Barcode Screen for Epigenetic Regulators Reveals a Role for the NuB4/HAT-B Histone Acetyltransferase Complex in Histone Turnover
Source: PLoS Genet. 2011 Oct 6;7(10):e1002284. doi: 10.1371/journal.pgen.1002284 (PMC3188528; doi:10.1371/journal.pgen.1002284)
Supplement: Table S4 — Primers used for deep sequencing. (DOC) [file pgen.1002284.s013.doc]

**TABLE S4: Primers used for deep sequencing**

| **Primer name** | **Primer sequence** |
| --- | --- |
| P7U2 | CAAGCAGAAGACGGCATACGAGATCGGCCATCAAAATGTATG |
| P7D2 | CAAGCAGAAGACGGCATACGAGATTTTTCGCCTCGACATCATCT |
| Seqi1U1 | ACACTCTTTCCCTACACGACGCTCTTCCGATCT**ATGC**GATGTCCACGAGGTCTCT |
| Seqi2U1 | ACACTCTTTCCCTACACGACGCTCTTCCGATCT**TACG**GATGTCCACGAGGTCTCT |
| Seqi3U1 | ACACTCTTTCCCTACACGACGCTCTTCCGATCT**GCAT**GATGTCCACGAGGTCTCT |
| Seqi4U1 | ACACTCTTTCCCTACACGACGCTCTTCCGATCT**CGTA**GATGTCCACGAGGTCTCT |
| Seqi5U1 | ACACTCTTTCCCTACACGACGCTCTTCCGATCT**ACTG**GATGTCCACGAGGTCTCT |
| Seqi6U1 | ACACTCTTTCCCTACACGACGCTCTTCCGATCT**CAGT**GATGTCCACGAGGTCTCT |
| Seqi7U1 | ACACTCTTTCCCTACACGACGCTCTTCCGATCT**TGAC**GATGTCCACGAGGTCTCT |
| Seqi8U1 | ACACTCTTTCCCTACACGACGCTCTTCCGATCT**GTCA**GATGTCCACGAGGTCTCT |
| Seqi9U1 | ACACTCTTTCCCTACACGACGCTCTTCCGATCT**AGCT**GATGTCCACGAGGTCTCT |
| Seqi1D1 | ACACTCTTTCCCTACACGACGCTCTTCCGATCT**ATGC**CGGTGTCGGTCTCGTAG |
| Seqi2D1 | ACACTCTTTCCCTACACGACGCTCTTCCGATCT**TACG**CGGTGTCGGTCTCGTAG |
| Seqi3D1 | ACACTCTTTCCCTACACGACGCTCTTCCGATCT**GCAT**CGGTGTCGGTCTCGTAG |
| Seqi4D1 | ACACTCTTTCCCTACACGACGCTCTTCCGATCT**CGTA**CGGTGTCGGTCTCGTAG |
| Seqi5D1 | ACACTCTTTCCCTACACGACGCTCTTCCGATCT**ACTG**CGGTGTCGGTCTCGTAG |
| Seqi6D1 | ACACTCTTTCCCTACACGACGCTCTTCCGATCT**CAGT**CGGTGTCGGTCTCGTAG |
| Seqi7D1 | ACACTCTTTCCCTACACGACGCTCTTCCGATCT**TGAC**CGGTGTCGGTCTCGTAG |
| Seqi8D1 | ACACTCTTTCCCTACACGACGCTCTTCCGATCT**GTCA**CGGTGTCGGTCTCGTAG |
| Seqi9D1 | ACACTCTTTCCCTACACGACGCTCTTCCGATCT**AGCT**CGGTGTCGGTCTCGTAG |
| P5seq | AATGATACGGCGACCACCGAGATCTACACTCTTTCCCTACACGACGCTCTTCCGATCT |
