# Supplementary material for: A Barcode Screen for Epigenetic Regulators Reveals a Role for the NuB4/HAT-B Histone Acetyltransferase Complex in Histone Turnover
Source: PLoS Genet. 2011 Oct 6;7(10):e1002284. doi: 10.1371/journal.pgen.1002284 (PMC3188528; doi:10.1371/journal.pgen.1002284)
Supplement: Table S5 — qPCR primers. (DOC) [file pgen.1002284.s014.doc]

**TABLE S5: qPCR primers**

| **Gene name** | **Primer name** | **Primer sequence** |
| --- | --- | --- |
| ADH1 | ADH1 PRO ii fwd | CCGTTGTTGTCTCACCATATCC |
| ADH1 | ADH1 PRO ii rev | GTTTCGTGTGCTTCGAGATACC |
| HHT2 | HHT2_QFor1 | GTGCCAAACGACCACAGTTG |
| HHT2 | HHT2_QRev1 | GGGCGTGCCAATAGTTTCAC |
| ADH2 | ADH2 PRO ii fwd | AACACCGGGCATCTCCAAC |
| ADH2 | ADH2 PRO ii rev | AAGTCGCTACTGGCACTC |
| IMD1 | QFORimd1 | TTTCGTGGGCTAGTACATTTTACCT |
| IMD1 | QREVimd1 | TGATAAGAAAAGTAAGGCAAGGAATAGA |
